# Bacillus thuringiensis Cry1Da_7 and Cry1B.868 Protein Interactions with Novel Receptors Allow Control of Resistant Fall Armyworms, Spodoptera frugiperda (J.E. Smith)

**DOI:** 10.1128/AEM.00579-19

**Published:** 2019-08-01

**Authors:** Yanfei Wang, Jinling Wang, Xiaoran Fu, Jeffrey R. Nageotte, Jennifer Silverman, Eric C. Bretsnyder, Danqi Chen, Timothy J. Rydel, Gregory J. Bean, Ke Sherry Li, Edward Kraft, Anilkumar Gowda, Autumn Nance, Robert G. Moore, Michael J. Pleau, Jason S. Milligan, Heather M. Anderson, Peter Asiimwe, Adam Evans, William J. Moar, Samuel Martinelli, Graham P. Head, Jeffrey A. Haas, James A. Baum, Fei Yang, David L. Kerns, Agoston Jerga

**Affiliations:** a Plant Biotechnology Program, Bayer Crop Science, Chesterfield, Missouri, USA; b Regulatory Science Program, Bayer Crop Science, Chesterfield, Missouri, USA; c Department of Entomology, Texas A&M University, College Station, Texas, USA; University of Bayreuth

**Keywords:** *Bacillus thuringiensis*, *Bt* toxin, Cry1B.868, Cry1Da_7, disabled insecticidal protein, insect resistance, mechanisms of action, mode of action

## Abstract

Two new modified Bacillus thuringiensis (*Bt*) proteins, Cry1Da_7 and Cry1B.868, with activity against fall armyworms (FAW), Spodoptera frugiperda (J.E. Smith), were evaluated for their potential to bind new insect receptors compared to proteins currently deployed as plant-incorporated protectants (PIPs) in row crops. Results from resistant insect bioassays, disabled insecticidal protein (DIP) bioassays, and cell-based assays using insect cells expressing individual receptors demonstrate that receptor utilizations of the newly modified Cry1Da_7 and Cry1B.868 proteins are distinct from each other and from those of commercially available *Bt* proteins such as Cry1F, Cry1A.105, Cry2Ab, and Vip3A. Accordingly, these two proteins target different insect proteins in FAW midgut cells and when pyramided together should provide durability in the field against this economically important pest.

**IMPORTANCE** There is increased concern with the development of resistance to insecticidal proteins currently expressed in crop plants, especially against high-resistance-risk pests such as fall armyworm (FAW), Spodoptera frugiperda, a maize pest that already has developed resistance to Bacillus thuringiensis (*Bt*) proteins such as Cry1F. Lepidopteran-specific proteins that bind new insect receptors will be critical in managing current Cry1F-resistant FAW and delaying future resistance development. Results from resistant insect assays, disabled insecticidal protein (DIP) bioassays, and cell-based assays using insect cells expressing individual receptors demonstrate that target receptors of the Cry1Da_7 and Cry1B.868 proteins are different from each other and from those of commercially available *Bt* proteins such as Cry1F, Cry1A.105, Cry2Ab, and Vip3A. Therefore, pyramiding these two new proteins in maize will provide durable control of this economically important pest in production agriculture.

## Abstract (precis)

There is increased concern with the development of resistance to insecticidal proteins currently expressed in crop plants, especially against high-resistance-risk pests such as fall armyworm (FAW), Spodoptera frugiperda, a maize pest that already has developed resistance to Bacillus thuringiensis (*Bt*) proteins such as Cry1F. Lepidopteran-specific proteins that bind new insect receptors will be critical in managing current Cry1F-resistant FAW and delaying future resistance development. Results from resistant insect assays, disabled insecticidal protein (DIP) bioassays, and cell-based assays using insect cells expressing individual receptors demonstrate that target receptors of the Cry1Da_7 and Cry1B.868 proteins are different from each other and from those of commercially available *Bt* proteins such as Cry1F, Cry1A.105, Cry2Ab, and Vip3A. Therefore, pyramiding these two new proteins in maize will provide durable control of this economically important pest in production agriculture.

## INTRODUCTION

Maize (Zea mays L. subsp. *mays*) is an important food source globally for both humans and animals. Since its introduction in Central America more than 7,000 years ago, its cultivation has spread across 175 million ha in over 20 countries by 2017 (1–3). The largest maize producers, the United States (33.84 million ha) and Brazil (17.55 million ha) (3), have quickly adopted improved crop protection practices, as almost a third of the attainable yield is lost due to pests, including 16% due to animal pests (4). This is especially important in Brazil and other tropical and subtropical environments, where insect pests such as fall armyworms (FAW), Spodoptera frugiperda, produce numerous generations per year on multiple host plants (5, 6), inflicting significant economic losses to farmers (7) and increasing the risk of insecticide resistance development (8). To mitigate these impacts on their harvests, farmers in the United States and Brazil use insect-protected (IP) maize that expresses Bacillus thuringiensis (*Bt*) proteins, comprising the majority of the total maize in cultivation (80% in the United States and 85% in Brazil) in 2017 (3). FAW are indigenous to the tropical regions of the Western Hemisphere and have recently been detected attacking maize plants in several sub-Saharan African countries as well as in Asia (9–15). Current IP maize products commercially available in Brazil and in Argentina to control FAW express one or combinations of distinct *Bt* proteins, including Cry1Ab, Cry1A.105, Cry1F, Cry2Ab, and Vip3A (16). Broad adaptation of a single insecticidal trait increases the risk of resistance development; FAW resistance to maize expressing Cry1F has been reported in Puerto Rico, Argentina, Brazil, and the United States (17–20). Cross-resistance to Cry1A proteins, including Cry1Ab, Cry1Ac, and Cry1A.105, has been observed for a colony isolated from the Puerto Rico population (21–24), underscoring the need to continue developing insecticidal proteins with new receptor preferences that are effective against this resistant insect. Several proteins from the Cry1B, Cry1C, and Cry1D subclasses are toxic to FAW (25, 26), and the commercial *Bt* formulation XenTari WG, containing Cry1C and Cry1D proteins, has been shown to control Cry1F-resistant FAW in diet bioassays (21, 26–29). This example demonstrates the potential value in deploying different Cry1 insecticidal protein subclasses in transgenic crops to overcome field resistance to other Cry1 subclasses primarily based on differences in receptor binding (30). Therefore, knowledge of the specific receptor utilization step in the overall mechanism of action (MOA) of pore-forming insecticidal proteins is a key component in deploying new IP maize products for increased durability (31–34). There are several methods used to study the receptor utilization of insecticidal proteins, including ligand blots (35, 36), *in vitro* binding experiments with labeled insecticidal proteins (30) and isolated insect gut brush border membrane vesicle (BBMV) preparations (37), pulldown experiments using immobilized or immunoprecipitated insecticidal proteins (38), insect cell-based assays using cloned insect receptor genes (39, 40), the disabled insecticidal protein (DIP) assay (41), and experiments with resistant insect colonies (42–45). Here, we report the development of two new modified insecticidal proteins for use against FAW, Cry1B.868 and Cry1Da_7, with enhanced specific activity against FAW and corn earworms (CEW), Helicoverpa zea (Boddie), respectively, and our comprehensive assessment of their FAW receptor preferences based on available resistant colonies, DIP assays, and cell-based receptor screens.

## RESULTS

### Identification of a Cry1B variant with improved toxicity toward FAW.

Chimeric Cry1B proteins exhibiting significantly higher insecticidal activities than the buffer negative control in bioassays at concentrations between 50 and 3,500 ng/cm^2^ were further characterized by bioassays following sucrose gradient purification of the crystalline inclusions, and the highest specific insecticidal activity was observed for Cry1B.868, which comprises domain 1 (D1) and domain 2 from Cry1Be2 (M1 to I503), domain 3 from Cry1Ca1 (N468 to N633), and the C-terminal protoxin moiety (domains 4 to 7) from Cry1Ab3 (E626 to E1155) (see Fig. S1 in the supplemental material). Cry1B.868 exerted mortality and developmental delay on FAW (and other lepidopteran insects [not shown]) at concentrations between 100 and 34,500 ng/cm^2^ (Fig. S2). Its MIC_50_ of 430 ng/cm^2^ against FAW using gradient-purified protein suggested an 8-fold improvement in specific activity toward this insect compared to the parental Cry1Be2 protein (Fig. S3).

### Identification of a Cry1Da variant with improved toxicity toward *Helicoverpa zea*.

Cry1Da.844_8 containing the S282V, Y316S, and I368P amino acid substitutions (Fig. S4) exhibited a 50-fold improvement in activity toward CEW (Fig. S5A) while maintaining toxicity toward FAW. The S282V, Y316S, and I368P mutations were also transferred back to the parent sequence, Cry1Da1, and the resulting variant was designated Cry1Da_7 (Fig. S4). The Cry1Da.844_8 and Cry1Da_7 proteins have 100% sequence identity in domains 1 to 3, and the full-length proteins were observed to readily undergo proteolysis using trypsin (data not shown). The resulting activated cores are comprised of the same pore-forming domain (D1) and same putative receptor biding domains (D2 and D3); correspondingly, their receptor utilizations were expected to be the same. The FAW activities of the Cry1Da_7 proteins with and without preproteolysis were comparable, suggesting that their PTX domains (domains 4 to 7) do not impact their stability or their receptor utilization in feeding assays (Fig. S5B). Consistent with this observation, the Cry1Da.844_8 and Cry1Da_7 proteins were also comparably active against FAW in insect feeding assays (Fig. S5C).

### Assessment of FAW-active insecticidal proteins against resistant FAW colonies.

The possibility of cross-resistance of the Cry1Fa-resistant FAW colony to the Cry1B.868 and Cry1Da_7 proteins was evaluated in comparative dose-response bioassays between susceptible (Cry1Fa-SS) and resistant (Cry1Fa-RR) colonies. There was no detectable difference in insect responses between the Cry1Fa-SS and Cry1Fa-RR colonies for both Cry1Da_7 and Cry1B.868, suggesting that the Cry1F-resistant colony was not cross-resistant to either Cry1Da_7 or Cry1B.868 (Table 1). Potential cross-resistance of Vip3A-resistant FAW to Cry1Da_7 and Cry1B.868 was also evaluated in comparative dose-response bioassays between susceptible (Vip3A-SS) and resistant (Vip3A-RR) colonies (46). Both Vip3A-SS and Vip3A-RR colonies were highly susceptible to the Cry1Da_7 protein, showing 50% lethal concentration (LC_50_) values of 11 and 12 ng/cm^2^, respectively (Table 2), and all insects were dead at concentrations above 316 ng/cm^2^ Cry1Da_7 (data not shown). Cry1B.868 was also highly toxic to the FAW colonies, with LC_50_ values of 262 ng/cm^2^ against the Vip3A-SS colony and 43 ng/cm^2^ against the Vip3A-RR colony (Table 2), indicating a lack of cross-resistance. Interestingly, the Vip3A-resistant colony was significantly more sensitive to this toxin than the Vip3A-susceptible colony; the data suggest that this difference is unrelated to the Vip3A resistance allele, and it is likely due to slight genetic background differences between the susceptible and resistant colonies.

**TABLE 1:** Comparative assessment of insecticidal proteins in insect feeding assays using susceptible and Cry1F-resistant fall armyworms, Spodoptera frugiperda

Sample	Dose^*a*^ (ng/cm^2^)	*S. frugiperda* colony^*b*^	Total no. of insect larvae	No. of larvae/repeat	Phenotypic distribution (%) (mean ± SD)^*c*^			
					Dead	1st instar	2nd instar	3rd instar
Buffer	0	SS	40	8	5.1 ± 11.1	0 ± 0	0 ± 0	94.9 ± 16.8
	0	Cry1Fa-RR	40	8	2.5 ± 5.6	0 ± 0	7.5 ± 6.8	90 ± 5.6
Cry1Da_7	690	SS	40	8	87.5 ± 12.5	12.5 ± 12.5	0 ± 0	0 ± 0
	690	Cry1Fa-RR	40	8	100 ± 0	0 ± 0	0 ± 0	0 ± 0
Cry1Da_7	6,900	SS	40	8	97.5 ± 5.6	2.5 ± 5.6	0 ± 0	0 ± 0
	6,900	Cry1Fa-RR	40	8	97.5 ± 5.6	2.5 ± 5.6	0 ± 0	0 ± 0
Cry1B.868	690	SS	40	8	97.5 ± 5.6	2.5 ± 5.6	0 ± 0	0 ± 0
	690	Cry1Fa-RR	40	8	80.0 ± 6.8	20.0 ± 6.8	0 ± 0	0 ± 0
Cry1B.868	6,900	SS	40	8	97.5 ± 5.6	2.5 ± 5.6	0 ± 0	0 ± 0
	6,900	Cry1Fa-RR	40	8	87.5 ± 12.5	12.5 ± 12.5	0 ± 0	0 ± 0
Cry1F.842	690	SS	40	8	90.0 ± 10.0	10.0 ± 10.0	0 ± 0	0 ± 0
	690	Cry1Fa-RR	40	8	0 ± 0	0 ± 0	2.5 ± 5.6	97.5 ± 5.6
Cry1F.842	6,900	SS	40	8	97.5 ± 5.6	2.5 ± 5.6	0 ± 0	0 ± 0
	6,900	Cry1Fa-RR	39	8	0 ± 0	0 ± 0	5.1 ± 7.0	94.9 ± 7.0

a Amount of protein sample per surface area of diet in a diet overlay assay.

b SS, Cry1Fa-susceptible insect colony; Cry1Fa-RR, Cry1Fa-resistant insect colony.

c Average phenotypic distribution of larvae across repeats.

**TABLE 2:** Comparative assessment of insecticidal proteins in insect feeding assays using susceptible and Vip3A-resistant fall armyworms, Spodoptera frugiperda

Sample	*S. frugiperda* colony^*a*^	No. of insects^*b*^	Mean slope ± SE	LC_50_ (ng/cm^2^) (95% CI)^*c*^	χ^2^	df^*d*^	Resistance ratio^*e*^
Cry1Da_7	SS	1,023	1.85 ± 0.18	12 (9–15)	13.36	26	1
	Vip3A-RR	1,088		11			−1.1
Cry1B.868	SS	1,021	3.69 ± 0.73	262 (192–363)	30.64	26	1
	Vip3A-RR	1,088	2.34 ± 0.16	43 (37–49)	16.77	30	−6.6
Vip3A	SS	1,024	1.85 ± 0.21	333 (237–475)	83.03	26	1
	Vip3A-RR	1,082		>31,600^*f*^			>94.8

a SS, Vip3A-susceptible insect colony; Vip3A-RR, Vip3A-resistant insect colony.

b Total number of insects evaluated.

c CI, confidence interval.

d df, degree of freedom.

e Calculated from the LC_50_ of the Vip3A-RR colony divided by the LC_50_ of the SS colony.

f Assumes that LC_50_ for Vip3A in the Vip3A-RR colony is >31,600 ng/cm^2^ (31,600 ng/cm^2^ is the highest dose tested).

### Construction of insecticidal protein variants disabled in their pore-forming function.

**FIG 1:**
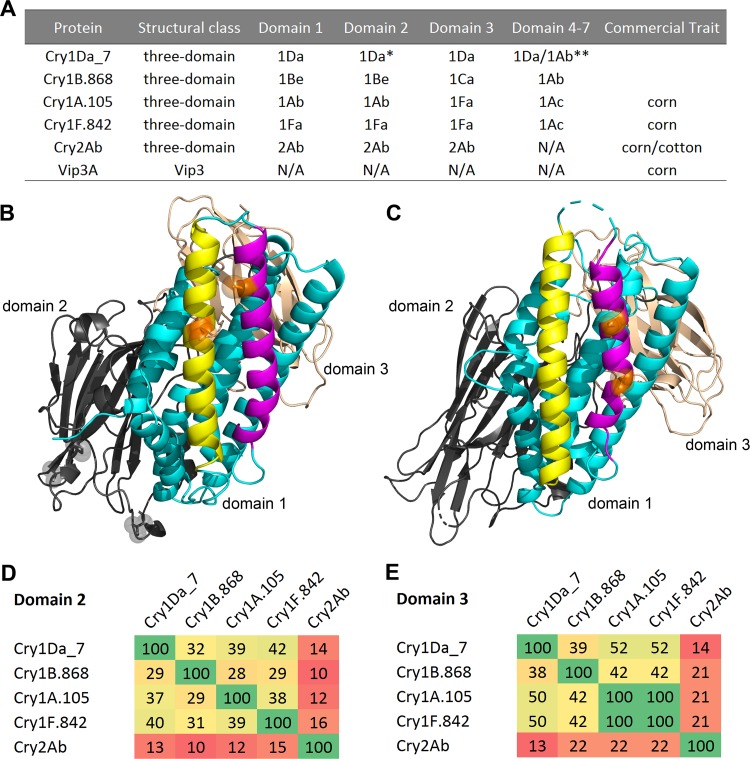
Sequence and structure relationship between *Bt* insecticidal proteins in current and next-generation above-ground traits. (A) Protein sequence information on the different NIPs, indicated by *Bt* toxin holotype nomenclature. The asterisk indicates the Cry1Da domain, in which substitutions were made to enhance CEW activity. Domains 4 to 7 of the three-domain Cry1 proteins are protoxin domains that are digested *in vivo* and thus are not part of the active ingredient; the Cry1Da_7 active core was appended to both Cry1Da and Cry1Ab protoxin domains and tested separately (double asterisk). Cry2Ab does not have these protoxin domains. Vip3A is of a different structural class whose sequence is different and structurally distinct from those of three-domain Cry proteins. N/A, not applicable. (B) Crystal structure of Cry1Da_7-DIP showing the three-domain architecture of domain 1 (cyan), domain 2 (gray), and domain 3 (light pink) in cartoon representation as well as helix 3 (yellow) and helix 4 (magenta) in domain 1. The key domain 1-disabling cysteine substitutions V108C and E128C are highlighted with orange sticks and semitransparent spheres corresponding to their side chain. The gray sticks and semitransparent spheres in domain 2 indicate the side chains of substitutions (S282V, Y316S, and I368P) that confer increased CEW specific activity. (C) Model of the three-dimensional architecture of Cry1B.868-DIP protein in cartoon representation with the above-described color scheme. The key domain 1-disabling substitutions A160N and N167D are highlighted with orange sticks and semitransparent spheres corresponding to their side chain. (D) Percent sequence identity between domains 2 of FAW-active insecticidal proteins based on comparative sequence analysis by multiple-sequence alignment (74). (E) Percent sequence identity between these proteins in domain 3.

DIP variants have been successfully developed to differentiate receptor preferences of Cry1Ab, Cry1Ca, and Cry2Ab proteins in *in vivo* binding studies based on introducing a substitution(s) of an amino acid(s) associated with the postbinding function of the protein, i.e., oligomerization and/or ion channel/pore formation, in domain 1 (41). We hypothesized that similar DIP probes can be developed for other three-domain endotoxins as well as for the Vip3A protein given the conserved ion channel/pore-forming function that is the recognized mode of action of these insecticidal toxins (Fig. 1) (41, 47–52). We implemented the same strategy to disable Cry1Da_7, Cry1B.868, and Cry1F.842 three-domain toxins. We targeted residues in helix 3 and helix 4 for substitution, and disruption of domain 1 function in the resulting variants was screened in insect feeding assays. We identified Cry1Da_7[V108C,E128C] (Fig. 1B), Cry1B.868[A160N,N167D] (Fig. 1C), and Cry1F.842[I108C,D128C] as DIP variants based on the selection criteria that these variants (i) had no significant insecticidal activity toward FAW (see Fig. 3), (ii) displayed similar processing with trypsin *in vitro* (data not shown), (iii) exhibited similar insecticidal activities in bioassays mixed with their native counterparts at a 1:1 molar concentration (see Fig. 3), and (iv) competed against their native counterparts in feeding assays with multiple lepidopteran species, including FAW, when presented in a molar excess of >10 (see Fig. 3). Substitutions in the Vip3A disabled toxin were made in the N-terminal domain of the protein, which is predicted to contain a series of α-helices similar to the reported structure of Escherichia coli hemolysin E, whereas the C-terminal putative receptor binding domains in Vip3A were unaltered (53, 54). It was also reported that Vip3A exists as a tetramer in solution before proteolysis and as an octameric complex comprised of heavy-chain (65-kDa) and light-chain (21-kDa) segments following serine protease treatment (55). We therefore hypothesized that this may be a prepore complex, and our disabled toxin design strategy was to cross-link these building blocks via four intermolecular disulfide bridges to restrict movement of these helices and membrane insertion. Comprehensive mutagenesis studies targeting the N-terminal domain of Vip3A identified Vip3Aa[S175C,L177C] as a DIP variant based on the above-described criteria.

### *In vivo* receptor binding assessment via competition assays between FAW-active insecticidal proteins and their DIP variants.

**FIG 2:**
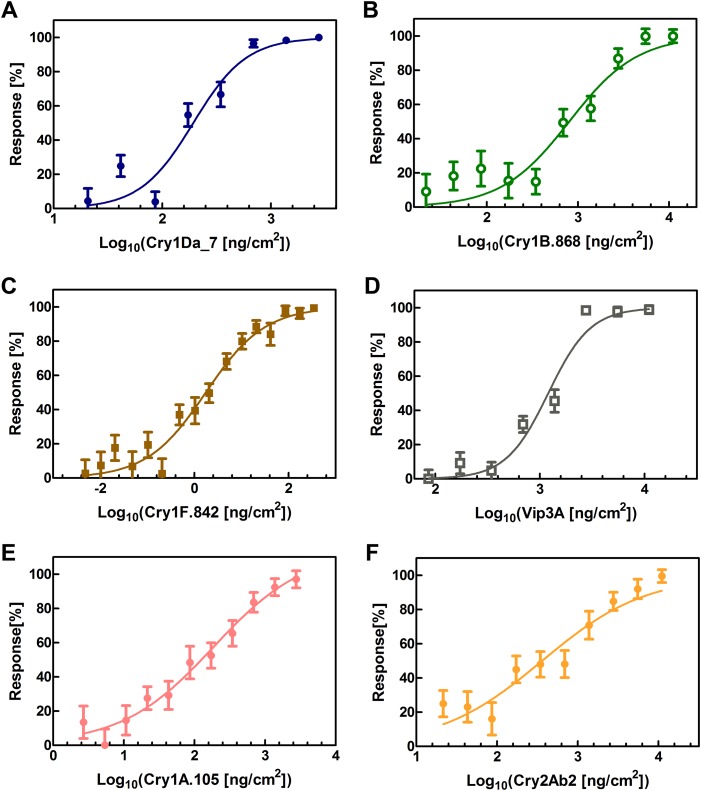
Dose-response assay of engineered insecticidal proteins in insect feeding assay using fall armyworms (FAW), Spodoptera frugiperda. Dose-response curves of Cry1Da_7 (A), Cry1B.868 (B), Cry1F.842 (C), Vip3A (D), Cry1A.105 (E), and Cry2Ab2 (F) show the mean insecticidal responses with standard errors as a function of the log_10_ value of the toxin dose. The insecticidal response was evaluated based on insect size with reference to the sizes of the positive control (100% response) and negative control (0% response), which were insects in the same assay treated with 2,760 ng/cm^2^ Cry1A.105 and buffer, respectively. See Materials and Methods for additional information.

**FIG 3:**
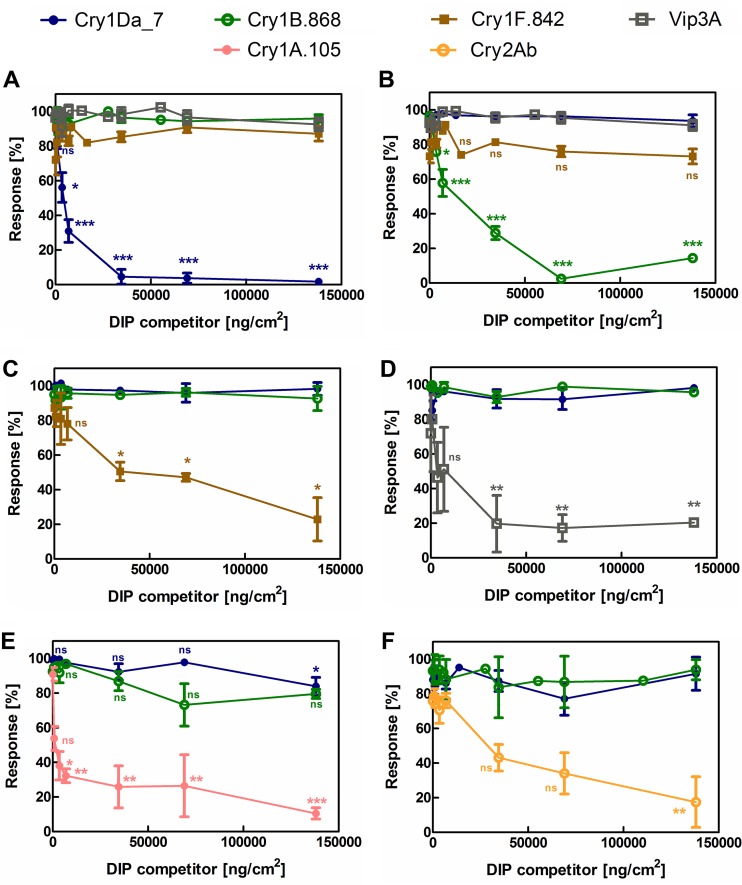
Homologous and heterologous DIP competition in insect feeding assays with FAW, Spodoptera frugiperda. DIP competition dose-response assays were performed by using a panel of native proteins, indicated by the symbols at the top, and the following competitor proteins: Cry1Da_7-DIP (A) Cry1B.868-DIP (B), Cry1F.842-DIP (C), Vip3A-DIP (D), Cry1A.105 (E), and Cry2Ab-DIP (F). The absolute DIP doses used are indicated on the *x* axis for each of the competition series, and the following fixed concentrations of NIPs were used: 690 ng/cm^2^ Cry1Da_7 (A to F), 5,520 ng/cm^2^ Cry1B.868 (A to F), 20.7 ng/cm^2^ Cry1F.842 (A to D), 2,760 ng/cm^2^ Vip3A (A to D), 690 ng/cm^2^ Cry1A.105 (E and F), and 2,760 ng/cm^2^ Cry2Ab2 (E and F). Statistical analyses of the data were done using multiple comparisons after ordinary one-way ANOVA and a Tukey *post hoc* test (α = 0.05); the symbols above the bars indicate these results with reference to buffer treatment (negative control), whereas symbols above the connector lines inform about differences between the connected treatment groups (ns, nonsignificant [*P* > 0.05]; *, *P* < 0.05; **, *P* < 0.01; ***, *P* < 0.001).

Cry1Da_7 at 690 ng/cm^2^ elicited a 98% insect-stunting response, which was calculated based on the observed insect size with reference to the sizes of the positive control (100% response) and negative control (0% response). DIP assays were then implemented to comparatively assess the receptor preferences of these native insecticidal proteins (NIPs). Cry1Da_7 at 690 ng/cm^2^ when coadministered with Cry1Da_7-DIP exhibited (i) no competition at stoichiometric DIP-to-NIP ratios, (ii) significant competition when DIP was used at a 5- to 25-fold excess of the NIP, and (iii) full competition when the DIP was used at a 50-fold excess of the NIP, where the insect phenotype was completely rescued and the insect size was indistinguishable from the size of the negative-control insects (see Fig. 3A). When Cry1Da_7-DIP was coadministered separately with 5,520 ng/cm^2^ Cry1B.868, 20.7 ng/cm^2^ Cry1F.842, and 2,760 ng/cm^2^ Vip3A (approximately an MIC_95_ dose) (Fig. 2), the insecticidal activity of these proteins was not inhibited, even in the presence of 138,000 ng/cm^2^ Cry1Da_7-DIP competitor, representing 25-, 6,600-, and 50-fold DIP-to-NIP challenge ratios, respectively (Fig. 3A). Similarly, homologous competition between NIPs and their corresponding DIP variants was demonstrated for Cry1B.868 (Fig. 3B), Cry1F.842 (Fig. 3C), Vip3A (Fig. 3D), Cry1A.105 (Fig. 3E), and Cry2Ab (Fig. 3F). Heterologous competition was also assessed between each NIP/DIP pair, and the insecticidal activity of Cry1Da_7, Cry1B.868, Cry1F.842, and Vip3A was not inhibited, even in the presence of a high concentration of the DIP competitor (Fig. 3A to D). We also evaluated these new insecticidal proteins against the disabled versions of two commercial insecticidal proteins, Cry1A.105 and Cry2Ab. Significant (*P* < 0.05) competition was not observed (Fig. 3E and F), with the exception of the comparison between 690 ng/cm^2^ Cry1Da_7 and 138,000 ng/cm^2^ Cry1A.105-DIP (Fig. 3E), which showed a mere 15% reduction of the insecticidal response under experimental conditions where Cry1A.105-DIP fully competed against its native counterpart.

### Receptor binding assessment using insect cell-based receptor screens.

**FIG 4:**
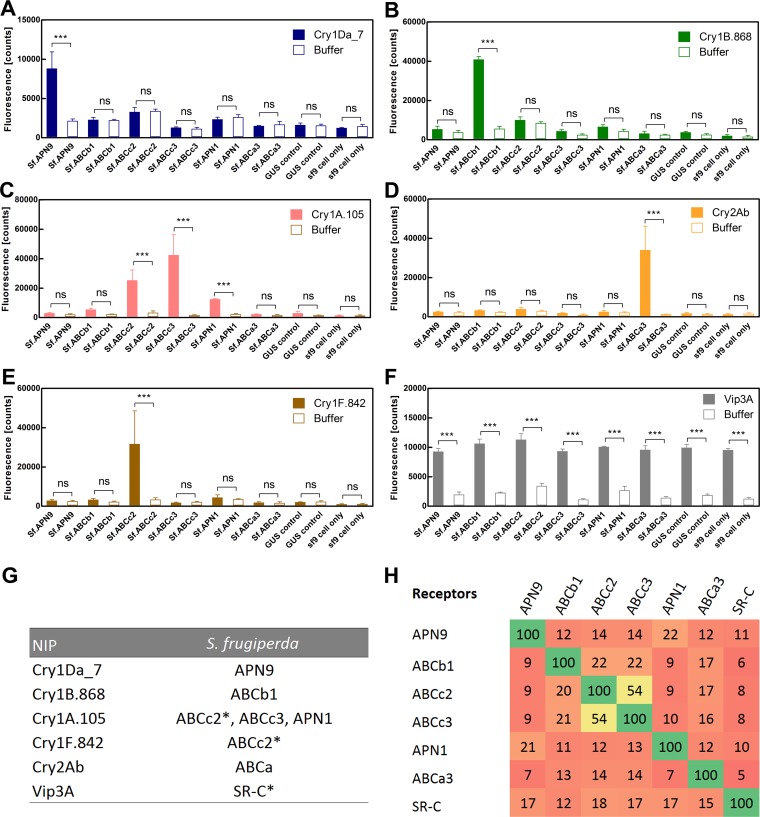
Receptor screen in a continuous cell line derived from FAW, Spodoptera frugiperda. (A to E) Receptor function was assessed by overexpressing insect receptors in Sf9 cells and incubating them with preactivated toxin. Cell permeability was assessed with SYTOX green nucleic acid stain based on literature precedent (71), and the mean fluorescence signals ± standard deviations were plotted for both the toxin-treated sample as well as the buffer-treated negative control: Cry1Da_7 (A), Cry1B.868 (B), Cry1F.842 (C), Cry2Ab (D), and Cry1A.105 (E). The specific insect proteins tested in Sf9 cells are indicated in the bar graph above the corresponding sample and negative control. Statistical analysis of the data was done using one-way ANOVA (α = 0.05); the symbols above the connector lines inform about differences between the connected treatment groups (ns, *P* > 0.05; *, *P* < 0.05; **, *P* < 0.01; ***, *P* < 0.001). (F) Summary of toxin-receptor pairs; the asterisk indicates FAW receptors whose toxin interactions *in vitro* and/or *in vivo* have been established (56, 63). (G) Percent sequence identity between the FAW proteins that act as functional receptors in cell-based assays. Comparative analysis of the amino acids sequences (see Fig. S6 in the supplemental material) was performed by multiple-sequence alignment (74).

Sf9 cells expressing the Sf.APN9 gene showed little to no SYTOX green fluorescence signal when buffer was added to the medium; however, robust fluorescence was observed in the presence of 50 μg/ml activated Cry1Da_7, indicating toxin-induced membrane permeabilization (Fig. 4A). Sf9 insect cells without Sf.APN9 expression did not show an increase in fluorescence (Fig. 4A), indicating that Sf.APN9 is a functional Cry1Da_7 receptor in cell-based assays. Cry1B.868 at 50 μg/ml elicited robust membrane permeabilization in Sf9 cells expressing Sf.ABCb1 (Fig. 4B), while only a background signal was observed when this receptor was not expressed (Fig. 4B). These results indicate that SfABCb1 and Cry1B.868 are a functional receptor-toxin pair. Additional cell-based receptor screens identified other interacting receptor-toxin pairs, including Sf.APN1/Cry1A.105 (Fig. 4C), Sf.ABCc2/Cry1A.105 (Fig. 4C), Sf.ABCc3/Cry1A.105 (Fig. 4C), Sf.ABCa3/Cry2Ab (Fig. 4D), and Sf.ABCc2/Cry1F.842 (Fig. 4E). Based on a report in the literature, Sf9 cell membrane permeabilization by Vip3A was also expected at a 50-μg/ml toxin dose due to the presence of its Sf.SC-R receptor (56). Consistent with this expectation, we observed this interaction between Vip3A and Sf9 cells (Fig. 4F). None of the three-domain toxins in our study showed such a response with the base cell line under these culturing conditions (Fig. 4A to E), suggesting that these Cry1 and Cry2 toxins do not interact with the Vip3A receptor, and they will not permeabilize Sf9 cells unless their corresponding receptor is overexpressed recombinantly.

### Proteomics assessment of FAW brush border membrane.

To assess whether the receptors identified in the insect cell-based screens were expressed in the FAW midgut, we isolated the brush border membrane (BBM) from FAW larval midgut and subjected BBM to bottom-up proteomics analysis (57). We confirmed the presence of Sf.APN9, Sf.ABCb1, Sf.ABCc2, Sf.ABCc3, Sf.ABCa3, and Sf.APN1 (Fig. S7).

## DISCUSSION

Discovering insecticidal proteins is critical for maintaining or increasing the durability of the next generation of IP trait-based products. This is especially true for high-resistance-risk insect pests such as FAW. The small number of currently available products targeting FAW implies that hundreds, if not thousands, of proteins need to be evaluated before finding a FAW-active protein that acts via a new receptor and, therefore, is capable of controlling field-evolved resistance. As there are numerous reports of a lack of cross-resistance between different subclasses of Cry1 proteins on FAW and other lepidopteran pests (30), we modified two subclasses of Cry1 proteins that have not yet been commercialized but have been reported to have FAW activity (25, 26). Our aim was to develop insecticidal proteins with improved insecticidal activity and with receptor preferences that are different from those of commercial *Bt* proteins. Our campaign identified two new modified insecticidal proteins, Cry1B.868 and Cry1Da_7, with enhanced specific activity against FAW and CEW, respectively. Cry1Da_7 and Cry1B.868 are both sufficiently sequence diverse (16) from each other and from current commercially available *Bt* proteins to support the hypothesis that they represent two new proteins that interact with different receptor proteins present in FAW. There have been numerous reports describing modification of *Bt* proteins to increase toxicity while maintaining specificity (58, 59). The Cry1Da_7 protein described here was identified by applying methods similar to those used for improving Cry51 against *Lygus* spp. (58). Cry1B.868 was developed using chimeragenesis, similar to the method used to develop the Cry1A.105 protein, a familiar protein that has been deployed commercially as *Bt* maize protected against FAW. As was previously demonstrated for Cry1A.105 and Cry51Aa2.834_16, the modifications resulting in Cry1Da_7 and Cry1B.868 should not alter their pore-forming mode of action as they undergo ingestion, solubilization, proteolytic activation, binding to specific midgut receptors, oligomerization, and pore formation that leads to cell lysis and, ultimately, mortality (60). This mode of action is shared with other commercialized Cry1, Cry2, and Cry3 proteins that are known to be specific and that have a long history of safe use. While various steps in the mode of action may contribute to the overall apparent specificity (61), recognition of receptors in the midgut is a significant contributing factor to overall specificity and susceptibility (30). Correspondingly, characterization of a Cry1F-resistant FAW population clearly demonstrates that the loss of a functional ABCc2 receptor from the midgut due to a defect in the *ABCC2* gene leads to a significant loss of Cry1F activity (62, 63). Also, mechanistic studies of the FAW insecticidal activity of Vip3Aa implicated the SR-C receptor interaction as a key step conferring Vip3Aa specificity (56). Cell-based assays demonstrated that both Cry1Da_7 and Cry1B.868 interact with different and very sequence-diverse (Fig. 4H) FAW receptor proteins compared to those identified for currently available commercial proteins. This suggests that loss, or modifications, of a receptor for a currently available commercial *Bt* protein would have very limited, if any, effect on the efficacy of Cry1Da_7 or Cry1B.868. Consistent with this hypothesis, FAW populations resistant to Cry1F and Vip3A were both at least as susceptible to Cry1Da_7 and Cry1B.868, as were comparable FAW populations that were susceptible to Cry1F and Vip3A. Interestingly, the Vip3A-resistant colony was significantly more sensitive to Cry1B.868 than the Vip3A-susceptible colony was; the data suggest that this difference is unrelated to the Vip3A resistance allele, and it is likely due to slight genetic background differences between the susceptible and resistant colonies. This is not without precedent, as a similar observation has been reported for Cry2Ab2 against Cry1Fa-resistant FAW (64).

Because we had access to resistant populations for only two of several relevant commercially available FAW-active proteins, we applied DIP assays to provide additional evidence that Cry1Da_7 and Cry1B.868 have unique receptor preferences. To validate that the observed reduction of insecticidal activity was due to competition between NIP and DIP for the limited insect receptor sites in the midgut, as opposed to other steps prerequisite for receptor binding (e.g., solubilization, reduction, and proteolytic activation) in the recognized *Bt* MOA model (61), we conducted DIP assays with presolubilized and trypsin-digested three-domain proteins and observed competition between each NIP-DIP pair (data not shown). Results from resistant insect assays, DIP assays, and cell-based assays demonstrate that receptor utilizations of Cry1Da_7 and Cry1B.868 proteins are significantly different from each other and from those of commercially available *Bt* proteins such as Cry1F.842, Cry1A.105, Cry2Ab, and Vip3A proteins. Taken together, these results support the conclusion that Cry1Da_7 and Cry1B.868 are effective insecticidal proteins against FAW that have the same pore-forming mode of action as other three-domain *Bt* proteins but bind to different receptors, suggesting that they would control resistance and increase product durability. Future experiments will have to be conducted to demonstrate the field efficacy of these proteins deployed as plant-incorporated protectants (PIPs) in row crops against FAW and other economically important lepidopteran species.

## MATERIALS AND METHODS

### Identification of the Cry1Da_7 variant with improved toxicity toward *Helicoverpa zea*.

A variant of Cry1Da1, designated Cry1Da.844, was selected as the parental protein for a campaign to improve toxicity toward CEW larvae. The Cry1Da.844 amino acid sequence (see Fig. S4A in the supplemental material) comprises the Cry1Da core toxin domain (M1 to K606) but utilizes the Cry1Ab3 protoxin domain (A623 to E1155) to ensure adequate expression in *Bt* and alkaline solubility of the parasporal crystals that are produced upon sporulation. The resulting coding region was cloned into a *Bt* expression vector and expressed as a crystal protein in the acrystalliferous *Bt* strain EG10650. A three-tiered optimization campaign employing both rational and statistically driven protein designs was used to identify Cry1Da.844 variants with significant improvements in toxicity toward CEW (3). Our rational protein design strategy was based on site saturation mutagenesis of amino acid residues in proximity to the putative receptor binding epitopes modeled via structural homology of Cry1Da to reported epitopes of Cry1A proteins (65, 66). The statistically driven protein design method was developed in-house and is similar to the previously reported protein sequence activity relationship (PROSAR) method (67). A combinatorial library was also generated from individual mutations identified as hits in the preliminary screens, and these variants were further screened in insect feeding assays to identify variants with more enhanced specific bioactivity.

### Construction of the chimeric protein Cry1B.868.

A series of chimeric Cry1B proteins was generated and screened for an expanded spectrum of insecticidal activity against lepidopteran pest species. DNA fragments encoding domains 1 and 2 of Cry1Be2 were fused to domain 3 fragments originating from a wide variety of three-domain Cry proteins. DNA sequences encoding the C-terminal half of the proteins extending beyond domain 3, and comprising the protoxin moiety, were derived from either Cry1Ab3 or Cry1Ac1. The resulting coding regions were cloned into a *Bt* expression vector and expressed as crystal proteins in the acrystalliferous *Bt* strain EG10650. Crystal proteins recovered from sporulated cultures were evaluated for toxicity against FAW.

### Protein preparation, cloning, and expression.

The proteins used in this study (Fig. 1) include the native insecticidal proteins Cry1Da, Cry1Da_7, Cry1D.844, Cry1D.844_8, Cry1B.868, Cry1A.105, Cry1F.842, Cry2Ab, and Vip3A as well as their disabled variants, Cry1D.844_8[V108C,E128C], Cry1B.868[A160N,N167D], Cry1B.867[A160N,N167D] (for X-ray crystallography only), Cry1F.842[I108C,D128C], Cry1A.105[I109C,E129C], Cry2Ab[R129Q,R139Q,G119C,N123A,L156C,R160A], and Vip3A[S175C,L177C]. All proteins, with the exception of the Vip3A proteins, were expressed in recombinant B. thuringiensis strains containing their respective expression plasmids. Single colonies from a glycerol stock of each of the *Bt* strains were isolated on Luria broth (LB) agar plates supplemented with chloramphenicol (5 μg/ml) at 30°C following overnight growth and used to inoculate 2.5-ml LB starter cultures containing chloramphenicol (3 μg/ml). Cells were grown at 25°C on a rotating roller drum overnight and then diluted into 500 ml *Bt* medium containing 3 μg/ml chloramphenicol in a 2-liter baffled flask and continued to grow at 20°C at 250 rpm for 65 h. Sporulation and crystal formation in the culture were verified by phase-contrast microscopy of a 2-μl aliquot of the *Bt* culture. Upon confirmation of the presence of crystals, the partially lysed sporulated cells were harvested by centrifugation at 4°C at 10,000 × *g* for 10 min. The pellet was then resuspended in 125 ml TX wash buffer containing 10 mM Tris at pH 7.5 and 0.005% Triton X-100 supplemented with 0.1 mM phenylmethylsulfonyl fluoride (PMSF), incubated at 250 rpm at 4°C for 15 min, and centrifuged again as described above. The resulting pellet was resuspended in 50 ml 1× phosphate-buffered saline (PBS) at pH 7.4 containing 0.1% Triton X-100, 2 mM MgCl_2_, and 10 U/ml Benzonase; incubated at 250 rpm at 4°C for 2 h; and centrifuged at 4°C at 10,000 × *g* for 10 min. Subsequently, the pellet, containing the spore-crystal mixture, was subjected to resuspension and centrifugation in the above-described TX buffer twice more. Expression of the Vip3A protein outfitted with an N-terminal His tag was conducted in Rosetta2(DE3) E. coli cells harboring the pET expression plasmid with the *vip3Aa1* gene ligated in. A large-scale ZYP-5052 autoinduction medium (75) supplemented with 100 μg/ml kanamycin and 25 μg/ml chloramphenicol was inoculated with cells, and the culture was stirred at 250 rpm at 18°C for 48 h. Cells were centrifuged; the cell pellet was resuspended in 50 ml lysis buffer containing a 3:1 (vol/vol) mixture of B-PER (bacterial protein extraction reagent; Thermo Scientific) and Y-PER (yeast protein extraction reagent; Thermo Scientific) supplemented with 0.1 mg/ml lysozyme, 5 μl Benzonase (ART.Sm nuclease at 1,125 U/μl, expressed from pMON101670), 1 tablet of an EDTA-free protease inhibitor cocktail (complete EDTA free, product number 11873580001; Roche), and 250 mM NaCl; and its pH was adjusted to pH 8.5. Cells were lysed while the mixture was stirred with a magnetic stir bar over a period of 30 min at 4°C. The homogenous mixture was then centrifuged at 20,000 × *g* at 4°C for 15 min using a FIBERLite F13-14x50cy rotor. The clean supernatant was transferred to a clean 50-ml Falcon tube and mixed with a 20-ml slurry of His-Select resin (Sigma), which was then gently rotated at 4°C for 30 min. The resin was loaded in a glass column outfitted with a glass frit and washed with 10 column volumes of buffer containing 20 mM Na-carbonate at pH 9, 300 mM NaCl, and 10 mM imidazole, followed by elution of the His tag protein in the same buffer supplemented with 500 mM imidazole. The eluted protein was concentrated and buffer exchanged into the above-described buffer without imidazole via diafiltration using Amicon Ultra centrifugal filters.

### Protein purification and activation.

Insecticidal proteins were solubilized from their respective spore-crystal mixtures in a buffer containing 100 ml 50 mM Na-carbonate at pH 11, 5 mM Tris(2-carboxyethyl)phosphine hydrochloride (TCEP), 1 mM PMSF, 1 mM EDTA, and 1 mM benzamidine over a period of 60 min while shaking at 250 rpm at 22°C. The insoluble debris was pelleted, and the full-length proteins were subjected to trypsinization and follow-up purification on a Q-Sepharose anion exchange column. Intact molecular weight determination using quadrupole time of flight liquid chromatography-mass spectrometry (Q-TOF LC-MS) provided the weight difference between full-length and truncated forms of the protein and was used to assess the N and C termini of the activated protein core (68). Spot densitometry using a bovine serum albumin (BSA) standard on SDS-PAGE gels was used to quantitate the protein samples. The activated samples were evaluated in cell-based assays (Fig. 4) as well as in DIP assays (data not shown).

### Insect bioassays.

Artificial-diet feeding assays were conducted with the following lepidopteran species: fall armyworm (FAW) (Spodoptera frugiperda) and corn earworm (CEW) (*Helicoverpa zea* Boddie). Insect eggs were obtained from Benzon Research (Carlisle, PA). Eggs for all lepidopteran assays were sourced 1 week before bioassays, and the cotton sheets containing insect eggs were stored at 15°C to 22°C until use. To hatch neonate insects, the cotton sheets were placed in a plastic Rubbermaid container with a moistened Kimwipe at the bottom to prevent excess drying of eggs. Neonates were hatched at 27°C overnight. Prior to infestation, the hatch box was cooled to 15°C to slow growth and deter clumping. CEW infestations were performed manually, and FAW infestations were performed using the entomology automated expansion (EAE) system, a modified flow cytometry system, to achieve a large fold increase in throughput. Insects were temporarily suspended in sheath solution containing 0.005% (wt/vol) Triton X-100 in distilled water and passed through the EAE system programmed to dispense one neonate insect in each well of 96-well diet microplates. These plates contained 200 μl molten Southland multiple-species diet with mold inhibitor in Serva agar, and they were treated with 20 μl of the protein sample, or a buffer control, via the surface contamination method. Following drying and infestation, the plates were sealed with preperforated heat seals and placed in an environmental chamber at 27°C with 60% relative humidity and a photoperiod of 14 h/10 h (light/dark). The size of FAW larvae was measured by an automated imaging system, and the insect stunting response was calculated based on the observed insect size with reference to the sizes of the positive control (100% response) and negative control (0% response); the positive control was Cry1A.105 at 3,450 ng/cm^2^, and the negative control was 20 mM sodium carbonate buffer. Toxin efficacy on CEW was evaluated manually based on insect mortality and instar stadium at day 5. Statistical analyses were performed using multiple comparisons after ordinary one-way analysis of variance (ANOVA) and a Tukey *post hoc* test (α = 0.05) using GraphPad Prism (GraphPad Software Inc.).

### *In vivo* receptor binding assessment via competition assays between FAW-active insecticidal proteins and their DIP variants.

To assess receptor binding preferences between the activated core of new and commercial insecticidal proteins on FAW, we implemented DIP assays (41) (Fig. 3) using the full-length spore-crystal preparations of Cry1Da_7 (with the Cry1Ab protoxin domain), Cry1B.868, Cry1F.842, Vip3A, Cry1A.105, Cry2Ab2, as well as a variant containing the disabling mutations. In these assays, a fixed concentration of NIP was premixed with increasing concentrations of DIP, and the resulting dilution series were administered to insects in a surface contamination feeding assay. A dose-response curve was first generated for each NIP in this study (Fig. 2) to assess the specific FAW activities based on 50% inhibitory concentration (IC_50_) calculations (Table 3) under assay conditions and to estimate the IC_95_ used as the fixed native IP concentration in DIP assays (45). If receptors are shared, then the inactive DIP probe saturates the receptors to which the NIP would ordinarily bind and acts as an antidote, whereas if there is no shared receptor, the toxicity of the NIP is not inhibited (41).

**TABLE 3:** Median inhibitory concentrations and 95% confidence limits based on larval size assessed in dose-response assays against fall armyworms, Spodoptera frugiperda

Protein	No. of insects^*a*^	Mean slope ± SE	IC_50_ (ng/cm^2^) (95% CI)^*b*^	sy.x^*c*^	df^*d*^	*R*^2^
Cry1Da_7	479	1.779 ± 0.242	193 (162–229)	44.49	477	0.42
Cry1B.868	399	1.165 ± 0.186	801 (599–1,073)	50.42	397	0.30
Cry1A.105	431	0.668 ± 0.334	186 (50–701)	48.79	427	0.30
Cry1F.842	321	0.694 ± 0.075	1.8 (1.3–2.4)	30.89	319	0.55
Cry2Ab	379	0.677 ± 0.089	361 (247–527)	46.44	377	0.26
Vip3A	504	2.149 ± 0.281	1,199 (1,050–1,370)	39.1	502	0.51

a Total number of insects evaluated.

b IC_50_, concentration necessary to reduce larval growth by 50%; CI, confidence interval.

c Standard deviation of the residuals calculated by GraphPad Prism.

d df, degree of freedom.

### Cry1Da_7 structure determination.

Only a purified sample of a construct which contained the mutations V108C, E128C, S282V, Y316S, and I368P relative to the wild-type protein yielded structure-solution-quality crystals. Crystal leads were sought via crystallization condition screening using a Phenix robot and 96-well crystal trays prefilled with commercially available crystallization condition screens. Cry1Da crystals resulted from the Wizard34 screen, condition G10 (20% polyethylene glycol 6000 [PEG 6000], 0.1 M morpholineethanesulfonic acid [MES] [pH 6] buffer, 0.2 M ammonium chloride). A 2.6-Å data set was collected remotely at the SER-CAT 22-BM beamline in the APS Synchrotron at Argonne National Laboratories. These data were reduced using the HKL package (76). The crystal was determined to have a trigonal/hexagonal lattice, with *a *=* b *=* *126.18 Å and *c *=* *126.22 Å, and angles of α = β = 90° and γ = 120°. The structure was solved by the molecular replacement method using the Phaser package (77) in CCP4i (78) with a previous 2.6-Å Cry1Ab-based structure. Successful structure solution revealed the true space group to be P3221. Refinement was performed using Refmac5 (79), and map fitting was done using Coot (80). The current structure of Cry1Da_7-DIP has an *R*_work_/*R*_free_ of 15.8%/20.7% for 41- to 2.6-Å (low- and high-resolution limit) data, and it extends from Leu28 to Ala593.

### Cry1B.868 structure model.

A purified sample of a disabled version of Cry1B.867 (containing mutations A160N and N167D) was crystallized using the JCSG^+^ screen, reagent A9 (20% PEG 3350, 0.2 M ammonium chloride). X-ray data were collected remotely at the SER-CAT 22-ID beamline, to obtain a 2.7-Å data set. HKL package analyses revealed the crystal to be hexagonal, with a lattice of *a *=* b *=* *106.18 Å and *c *=* *85.30 Å and angles of α=β = 90° and γ = 120°. The structure was solved by the molecular replacement method using the Phaser package (77) in CCP4i (78), using the Cry8Ea1 structure under PDB accession number 3EB7 for phasing. Successful structure solution revealed the true space group to be P63. Refinement was performed using Refmac5 (79), and map fitting was done using Coot (80). The current structure of the Cry1B.867-DIP variant has an *R*_work_/*R*_free_ of 22.3%/27.7% for 38- to 2.7-Å data, and it extends from Ser54 to Thr640. The Cry1B.868 model was generated by the chainsaw utility in CCP4 using the PDB coordinates of the three-dimensional crystal structure of Cry1B.867 and the primary amino acid sequence of the highly homologous protein Cry1B.868 (69).

### Spodoptera frugiperda insect cell assays.

Sf9 insect cells (Life Technologies), originally derived from ovarian cells of Spodoptera frugiperda (70), were used to assess receptor function in cell-based toxicity assays (71). The cells were plated in 100 μl Sf-900 III serum-free insect cell culture medium (Life Technologies) at a density of 50,000 cells and 1 μl of P3 or P4 baculovirus stocks in each well of a 96-well optical-bottom black culture plate (Nunc; Thermo Scientific). The P3 or P4 baculovirus stocks encode the receptor sequences reported in this study. The plates were kept in a humidified environment to prevent evaporation and incubated at 27°C for 48 h. Receptor expression was confirmed by Western blotting. Toxins were diluted to the same protein concentration (50 μg/ml) in unsupplemented Grace’s insect medium with 2 μM SYTOX green nucleic acid stain (catalog number S7020; Life Technologies). The medium was removed from the wells without disturbing the attached cells, and the diluted toxins or buffer controls were added in the corresponding wells. The fluorescence intensity was measured on a CLARIOstar microplate reader (BMG Labtech) after incubation at 27°C for 4 h. The data from technical replicates were averaged, and the mean signals as well as the corresponding standard deviations were plotted for each receptor condition with and without insecticidal protein addition. Statistical analyses were performed using multiple comparisons after ordinary one-way ANOVA and a Tukey *post hoc* test (α = 0.05) using GraphPad Prism (GraphPad Software Inc.).

### Brush border membrane preparation for mass spectrometry.

The brush border membrane (BBM) or microvillar membrane was prepared using third-instar whole FAW larvae via the cation differential precipitation method using 10 mM calcium chloride, which was a modified version of the procedure implemented for Pieris brassicae (L.) by Wolfersberger et al. (72). The total protein concentration in both the BBM sample and the initial insect homogenate was determined by a Bradford assay (Bio-Rad) according to the manufacturer’s protocol, and the quality of the BBM preparation was evaluated based on a partial biochemical characterization measuring specific alkaline phosphatase (ALP) and leucine aminopeptidase (APN) (73) enzyme activities of both the BBM fraction and the initial insect homogenate.

### Proteomics analysis.

BBM samples containing a total of 50 μg proteins were reduced and subjected to microwave-assisted trypsinization by Discover Proteomics (CEM, Matthews, NC, USA). The proteolysis reaction was terminated by adding trifluoroacetic acid to a final concentration of 1% (vol/vol). The sample was centrifuged at 21,000 × *g* for 30 min at room temperature to pellet the BBM. The supernatant was collected and subjected to proteomics analysis. For separation, the peptides were injected into an Ultimate 3000 nano-LC system equipped with an inline reverse-phase C_18_ trap column (PepMap, 300-μm internal diameter [ID] by 5 mm; Thermo Fisher Scientific, Waltham, MA, USA) and a high-resolution C_18_ Acclaim PepMap reverse-phase liquid chromatography column (75-μm ID by 150 mm; Thermo Fisher Scientific). The peptides were loaded onto the trap column at a flow rate of 5 μl/min using 0.1% formic acid in water and eluted at a flow rate of 300 nl/min using a binary mobile phase comprised of 0.1% formic acid in water and a linear gradient of acetonitrile between 10 and 30% (vol/vol) over 40 min. The eluted peptides were injected into the nanospray of a Q-Exactive HF Orbitrap mass spectrometer (Thermo Fisher Scientific, Waltham, MA, USA) operating in full data-dependent tandem mass spectrometry mode. The nanospray voltage was kept at 1.9 kV, and data were collected using Xcalibur software (Thermo Fisher Scientific, Waltham, MA, USA). Full-scan mass spectra were acquired with the Orbitrap instrument over a mass range of *m/z* 400 to 1,600, with a resolution of 120,000 (*m/z* 400) and an automatic gain control (AGC) target of 3 × 10^6^. A lock mass function was used to obtain high mass accuracy. The 12 most intense precursor ions were selected for collision-induced fragmentation, with a normalized collision energy of 27%, a resolution of 15,000, and an AGC target of 1 × 10^5^. For each sample, the injection volume was adjusted per the protein assay to load 1 μg onto the column. Experiments for each sample were done in three technical replicates. Proteins were identified by Proteome Discoverer (version 1.4; Thermo Fisher Scientific, Waltham, MA, USA). The FAW protein database (1,786 sequences) from UniProt was combined with the FAW APN1, APN9, ABCa3, ABCb1, ABCc2, ABCc3, and SR-C protein sequences, and a reversed decoy database was used for comparison. Data files were generated from acquired raw data files with Thermo Xcalibur. The protein identifications were filtered in Proteome Discoverer, retaining only proteins that contained at least three peptides with XCorr (cross-correlation value in Proteome Discoverer) scores above the threshold. The data include only rank 1 peptides and peptides in the top-scored proteins. Trypsin was specified as the proteolytic enzyme, and one missed cleavage was allowed. Peptide mass tolerance was set at 10 ppm, fragment mass tolerance was set at 0.6 Da, and peptide charge was set at +2, +3, and +4. False discovery rates for peptide identification of all searches were less than 5.0%.

### Data availability.

The current structure of Cry1Da_7-DIP was deposited in the RCSB Protein Data Bank under accession number 6OVB. The current structure of the Cry1B.867-DIP variant was deposited in the RCSB Protein Data Bank under accession number 6OWK.

## Supplementary Material

Supplemental file 1
