# Supplementary material for: Bacillus thuringiensis Cry1Da_7 and Cry1B.868 Protein Interactions with Novel Receptors Allow Control of Resistant Fall Armyworms, Spodoptera frugiperda (J.E. Smith)
Source: Appl Environ Microbiol. 2019 Aug 1;85(16):e00579-19. doi: 10.1128/AEM.00579-19 (PMC6677855; doi:10.1128/AEM.00579-19)
Supplement: Supplemental file 1 [file AEM.00579-19-s0001.pdf]

Novel receptor interaction of *Bacillus thuringiensis* Cry1Da_7 and Cry1B.868 proteins allow control of resistant fall armyworm, *Spodoptera frugiperda* (J.E. Smith)

Yanfei Wang[1], Jinling Wang[1], Xiaoran Fu[1], Jeffrey R. Nageotte[1], Jennifer Silverman[1], Eric C. Bretsnyder[1], Danqi Chen[1], Timothy J. Rydel[1], Gregory J. Bean[1], Ke Sherry Li[1], Edward Kraft[1], Anilkumar Gowda[2], Autumn Nance[1], Robert G. Moore[1], Michael J. Pleau[1], Jason S. Milligan[1], Heather M. Anderson[2], Peter Asiimwe[2], Adam Evans[1], William J. Moar[2], Samuel Martinelli[2], Graham P. Head[2], Jeffrey A. Haas[1], James A. Baum[1], Fei Yang[3], David L. Kerns[3], and Agoston Jerga[1]

[1]From the Plant Biotechnology Program, Bayer Crop Science, Chesterfield, MO 63017

[2]From the Regulatory Science Program, Bayer Crop Science, Chesterfield, MO 63017

[3]From the Department of Entomology, Texas A&M University, Entomology Research Laboratory 103J, College Station, TX 77843-2475

*Running title: New insecticidal proteins to combat *Bt*-resistant fall armyworm

To whom correspondence should be addressed: Agoston Jerga, GG4448-A, Bayer Crop Science, 700 Chesterfield Parkway West, Chesterfield, MO 63017, USA, Tel.: (636)737-7139; E-mail: agoston.jerga@bayer.com

Supplementary Figure 1.

A Cry1B.868

```
   1 MTSNRKNENE IINALSIPAV SNHSAQMNLS TDARIEDSLC IAEGNNIDPF
  51 VSASTVQTGI NIAGRILGVL GVPFAGQIAS FYSFLVGELW PRGRDPWEIF
 101 LEHVEQLIRQ QVTENTRDTA LARLQGLGNS FRAYQQSLED WLENRDDART
 151 RSVLYTQYIA LELDFLNAMP LFAIRNQEVP LLMVYAQAAN LHLLLLRDAS
 201 LFGSEFGLTS QEIQRYYERQ VEKTREYSDY CARWYNTGLN NLRGTNAESW
 251 LRYNQFRRDL TLGVLDLVAL FPSYDTRVYP MNTSAQLTRE IYTDPIGRTN
 301 APSGFASTNW FNNNAPSFSA IEAAVIRPPH LLDFPEQLTI FSVLSRWSNT
 351 QYMNYWVGHR LESRTIRGSL STSTHGNTNT SINPVTLQFT SRDVYRTESF
 401 AGINILLTTP VNGVPWARFN WRNPLNSLRG SLLYTIGYTG VGTQLFDSET
 451 ELPPETTERP NYESYSHRLS NIRLISGNTL RAPVYSWTHR SADRTNTISS
 501 DSINQIPLVK GFRVWGGTSV ITGPGFTGGD ILRRNTFGDF VSLQVNINSP
 551 ITQRYRLRFR YASSRDARVI VLTGAASTGV GGQVSVNMPL QKTMEIGENL
 601 TSRTFRYTDF SNPFSFRANP DIIGISEQPL FGAGSISSGE LYIDKIEIIL
 651 ADATFEAESD LERAQKAVNE LFTSSNQIGL KTDVTDYHID QVSNLVECLS
 701 DEFCLDEKKE LSEKVKHAKR LSDERNLLQD PNFRGINRQL DRGWRGSTDI
 751 TIQGGDDVFK ENYVTLLGTF DECYPTYLYQ KIDESKLKAY TRYQLRGYIE
 801 DSQDLEIYLI RYNAKHETVN VPGTGSLWPL SAPSPIGKCA HHSHHFSLDI
 851 DVGCTDLNED LGVWVIFKIK TQDGHARLGN LEFLEEKPLV GEALARVKRA
 901 EKKWRDKREK LEWETNIVYK EAKESVDALF VNSQYDRLQA DTNIAMIHAA
 951 DKRVHSIREA YLPELSVIPG VNAAIFEELE GRIFTAFSLY DARNVIKNGD
1001 FNNGLSCWNV KGHVDVEEQN NHRSVLVVPE WEAEVSQEVR VCPGRGYILR
1051 VTAYKEGYGE GCVTIHEIEN NTDELKFSNC VEEEVYPNNT VTCNDYTATQ
1101 EEYEGTYTSR NRGYDGAYES NSSVPADYAS AYEEKAYTDG RRDNPCESNR
1151 GYGDYTPLPA GYVTKELEYF PETDKVWIEI GETEGTFIVD SVELLLMEE
```

B Cry1Be2

```
   1 MTSNRKNENE IINALSIPAV SNHSAQMNLS TDARIEDSLC IAEGNNIDPF
  51 VSASTVQTGI NIAGRILGVL GVPFAGQIAS FYSFLVGELW PRGRDPWEIF
 101 LEHVEHLIRQ QVTENTRDTA LARLQGLGNS FRAYQQSLED WLENRDDART
 151 RSVLYTQYIA LELDFLNAMP LFAIRNQEVP LLMVYAQAAN LHLLLLRDAS
 201 LFGSEFGLTS QEIQRYYERQ VEKTREYSDY CARWYNTGLN NLRGTNAESW
 251 LRYNQFRRDL TLGVLDLVAL FPSYDTRVYP MNTSAQLTRE IYTDPIGRTN
 301 APSGFASTNW FNNNAPSFSA IEAAVIRPPH LLDFPEQLTI FSVLSRWSNT
 351 QYMNYWVGHR LESRTIRGSL STWTHGNTNT SINPVTLQFT SRDVYRTESF
 401 AGINILLTTP VNGVPWARFN WRNPLNSLRG SLLYTIGYTG VGTQLFDSET
 451 ELPPETTERP NYESYSHRLS NIRLISGNTL RAPVYSWTHR SADRTNTISS
 501 DSITQIPLVK SFNLNSGTSV VSGPGFTGGD IIRTNVNGSV LSMGLNFNNT
 551 SLQRYRVRVR YAASQTMVLR VTVGGSTTFD QGFPSTMSAN ESLTSQSFRF
 601 AEFPVGISAS GSQTAGISIS NNAGRQTFHF DKIEFIPITA TFEAEYDLER
 651 AQEAVNALFT NTNPRRLKTG VTDYHIDEVS NLVACLSDEF CLDEKRELLE
 701 KVKYAKRLSD ERNLLQDPNF TSINKQPDFN SNNEQSNFTS IHEQSEHGWW
 751 GSENITIQEG NDVFKENYVT LPGTFNECYP TYLYQKIGEA ELKAYTRYQL
 801 SGYIEDSQDL EIYLIRYNAK HETLDVPGTE SVWPLSVESP IGRCGEPNRC
 851 APHFEWNPDL DCSCRDGEKC AHHSHHFSLD IDVGCIDLHE NLGVWVVFKI
 901 KTQEGHARLG NLEFIEEKPL LGEALSRVKR AEKKWRDKRE KLQLETKRVY
 951 TEAKEAVDAL FVDSQYDRLQ ADTNIGMIHA ADKLVHRIRE AYLSELSVIP
1001 GVNAEIFEEL EGRIITAISL YDARNVVKNG DFNNGLACWN VKGHVDVQQS
1051 HHRSVLVIPE WEAEVSQAVR VCPGRGYILR VTAYKEGYGE GCVTIHEIEN
1101 NTDELKFKNC EEEEVYPTDT GTCNDYTAHQ GTAVCNSRNA GYEDAYEVDT
1151 TASVNYKPTY EEETYTDVRR DNHCEYDRGY VNYPPVPAGY MTKELEYFPE
1201 TDKVWIEIGE TEGKFIVDSV ELLLMEE
```

C Cry1Ca1

```
   1 MEENNQNQCI PYNCLSNPEE VLLDGERIST GNSSIDISLS LVQFLVSNFV
  51 PGGGFLVGLI DFVWGIVGPS QWDAFLVQIE QLINERIAEF ARNAAIANLE
 101 GLGNNFNIYV EAFKEWEEDP NNPETRTRVI DRFRILDGLL ERDIPSFRIS
 151 GFEVPLLSVY AQAANLHLAI LRDSVIFGER WGLTTINVNE NYNRLIRHID
 201 EYADHCANTY NRGLNNLPKS TYQDWITYNR LRRDLTLTVL DIAAFFPNYD
 251 NRRYPIQPVG QLTREVYTDP LINFNPQLQS VAQLPTFNVM ESSRIRNPHL
 301 FDILNNLTIF TDWFSVGRNF YWGGHRVISS LIGGGNITSP IYGREANQEP
 351 PRSFTFNGPV FRTLSNPTLR LLQQPWPAPP FNLRGVEGVE FSTPTNSFTY
 401 RGRGTVDSLT ELPPEDNSVP PREGYSHRLC HATFVQRSGT PFLTTGVVFS
 451 WTDRSATLTN TIDPERINQI PLVKGFRVWG GTSVITGPGF TGGDILRRNT
 501 FGDFVSLQVN INSPITQRYR LRFRYASSRD ARVIVLTGAA STGVGGQVSV
 551 NMPLQKTMEI GENLTSRTFR YTDFSNPFSF RANPDIIGIS EQPLFGAGSI
 601 SSGELYIDKI EIILADATFE AESDLERAQK AVNALFTSSN QIGLKTDVTD
 651 YHIDQVSNLV DCLSDEFCLD EKRELSEKVK HAKRLSDERN LLQDPNFRGI
 701 NRQPDRGWRG STDITIQGGD DVFKENYVTL PGTVDECYPT YLYQKIDESK
 751 LKAYTRYELR GYIEDSQDLE IYLIRYNAKH EIVNVPGTGS LWPLSAQSPI
 801 GKCGEPNRCA PHLEWNPDLD CSCRDGEKCA HHSHHFTLDI DVGCTDLNED
 851 LGVWVIFKIK TQDGHARLGN LEFLEEKPLL GEALARVKRA EKKWRDKREK
 901 LQLETNIVYK EAKESVDALF VNSQYDRLQV DTNIAMIHAA DKRVHRIREA
 951 YLPELSVIPG VNAAIFEELE GRIFTAYSLY DARNVIKNGD FNNGLLCWNV
1001 KGHVDVEEQN NHRSVLVIPE WEAEVSQEVR VCPGRGYILR VTAYKEGYGE
1051 GCVTIHEIED NTDELKFSNC VEEEVYPNNT VTCNNYTGTQ EEYEGTYTSR
1101 NQGYDEAYGN NPSVPADYAS VYEEKSYTDG RRENPCESNR GYGDYTPLPA
1151 GYVTKDLEYF PETDKVWIEI GETEGTFIVD SVELLLMEE
```

D Cry1Ab3

```
   1 MDNNPNINEC IPYNCLSNPE VEVLGGERIE TGYTPIDISL SLTQFLLSEF
  51 VPGAGFVLGL VDIIWGIFGP SQWDAFLVQI EQLINQRIEE FARNQAISRL
 101 EGLSNLYQIY AESFREWEAD PTNPALREEM RIQFNDMNSA LTTAIPLFAV
 151 QNYQVPLLSV YVQAANLHLS VLRDVSVFGQ RWGFDAATIN SRYNDLTRLI
 201 GNYTDHAVRW YNTGLERVWG PDSRDWIRYN QFRRELTLTV LDIVSLFPNY
 251 DSRTYPIRTV SQLTREIYTN PVLENFDGSF RGSAQGIEGS IRSPHLMDIL
 301 NSITIYTDAH RGEYYWSGHQ IMASPVGFSG PEFTFPLYGT MGNAAPQQRI
 351 VAQLGQGVYR TLSSTLYRRP FNIGINNQQL SVLDGTEFAY GTSSNLPSAV
 401 YRKSGTVDSL DEIPPQNNNV PPRQGFSHRL SHVSMFRSGF SNSSVSIIRA
 451 PMFSWIHRSA EFNNIIPSSQ ITQIPLTKST NLGSGTSVVK GPGFTGGDIL
 501 RRTSPGQIST LRVNITAPLS QRYRVRIRYA STTNLQFHTS IDGRPINQGN
 551 FSATMSSGSN LQSGSFRTVG FTTPFNFSNG SSVFTLSAHV FNSGNEVYID
 601 RIEFVPAEVT FEAEYDLERA QKAVNELFTS SNQIGLKTDV TDYHIDQVSN
 651 LVECLSDEFC LDEKKELSEK VKHAKRLSDE RNLLQDPNFR GINRQLDRGW
 701 RGSTDITIQG GDDVFKENYV TLLGTFDECY PTYLYQKIDE SKLKAYTRYQ
 751 LRGYIEDSQD LEIYLIRYNA KHETVNVPGT GSLWPLSAPS PIGKCAHHSH
 801 HFSLDIDVGC TDLNEDLGVW VIFKIKTQDG HARLGNLEFL EEKPLVGEAL
 851 ARVKRAEKKW RDKREKLEWE TNIVYKEAKE SVDALFVNSQ YDRLQADTNI
 901 AMIHAADKRV HSIREAYLPE LSVIPGVNAA IFEELEGRIF TAFSLYDARN
 951 VIKNGDFNNG LSCWNVKGHV DVEEQNNHRS VLVVPEWEAE VSQEVRVCPG
1001 RGYILRVTAY KEGYGEGCVT IHEIENNTDE LKFSNCVEEE VYPNNTVTCN
1051 DYTATQEEYE GTYTSRNRGY DGAYESNSSV PADYASAYEE KAYTDGRRDN
1101 PCESNRGYGD YTPLPAGYVT KELEYFPETD KVWIEIGETE GTFIVDSVEL
1151 LLMEE
```

Supplementary Figure 2.

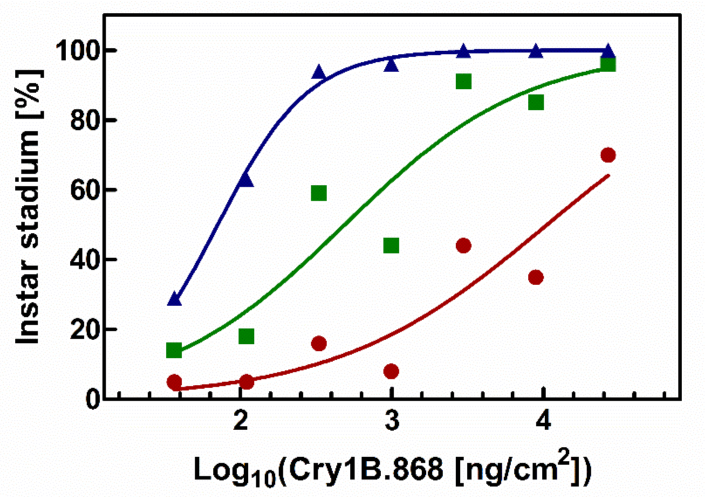

Supplementary Figure 3.

Protein	Slope	MIC$_{50}$ (µg/cm2)	95% CI
Cry1Be2	1.55	3.23	2.16 – 5.06
Cry1B.868	1.63	0.43	0.28 – 0.62

Supplementary Figure 4.

A

Cry1Da.844

```
   1 MEINNQNQCV PYNCLSNPKE IILGEERLET GNTVADISLG LINFLYSNFV
  51 PGGGFIVGLL ELIWGFIGPS QWDIFLAQIE QLISQRIEEF ARNQAISRLE
 101 GLSNLYKVYV RAFSDWEKDP TNPALREEMR IQFNDMNSAL ITAIPLFRVQ
 151 NYEVALLSVY VQAANLHLSI LRDVSVFGER WGYDTATINN RYSDLTSLIH
 201 VYTNHCVDTY NQGLRRLEGR FLSDWIVYNR FRRQLTISVL DIVAFFPNYD
 251 IRTYPIQTAT QLTREVYLDL PFINENLSPA ASYPTFSAAE SAIIRSPHLV
 301 DFLNSFTIYT DSLARYAYWG GHLVNSFRTG TTTNLIRSPL YGREGNTERP
 351 VTITASPSVP IFRTLSYITG LDNSNPVAGI EGVEFQNTIS RSIYRKSGPI
 401 DSFSELPPQD ASVSPAIGYS HRLCHATFLE RISGPRIAGT VFSWTHRSAS
 451 PTNEVSPSRI TQIPWVKAHT LASGASVIKG PGFTGGDILT RNSMGELGTL
 501 RVTFTGRLPQ SYYIRFRYAS VANRSGTFRY SQPPSYGISF PKTMDAGEPL
 551 TSRSFAHTTL FTPITFSRAQ EEFDLYIQSG VYIDRIEFIP VTATFEAEYD
 601 LERAQKAVNE LFTSSNQIGL KTDVTDYHID QVSNLVECLS DEFCLDEKKE
 651 LSEKVKHAKR LSDERNLLQD PNFRGINRQL DRGWRGSTDI TIQGGDDVFK
 701 ENYVTLLGTF DECYPTYLYQ KIDESKLKAY TRYQLRGYIE DSQDLEIYLI
 751 RYNAKHETVN VPGTGSLWPL SAPSPIGKCA HHSHHFSLDI DVGCTDLNED
 801 LGVWVIFKIK TQDGHARLGN LEFLEEKPLV GEALARVKRA EKKWRDKREK
 851 LEWETNIVYK EAKESVDALF VNSQYDRLQA DTNIAMIHAA DKRVHSIREA
 901 YLPELSVIPG VNAAIFEELE GRIFTAFSLY DARNVIKNGD FNNGLSCWNV
 951 KGHVDVEEQN NHRSVLVVPE WEAEVSQEVR VCPGRGYILR VTAYKEGYGE
1001 GCVTIHEIEN NTDELKFSNC VEEEVYPNNT VTCNDYTATQ EEYEGTYTSR
1051 NRGYDGAYES NSSVPADYAS AYEEKAYTDG RRDNPCESNR GYGDYTPLPA
1101 GYVTKELEYF PETDKVWIEI GETEGTFIVD SVELLLMEE
```

B

Cry1Da1

```
   1 MEINNQNQCV PYNCLSNPKE IILGEERLET GNTVADISLG LINFLYSNFV
  51 PGGGFIVGLL ELIWGFIGPS QWDIFLAQIE QLISQRIEEF ARNQAISRLE
 101 GLSNLYKVYV RAFSDWEKDP TNPALREEMR IQFNDMNSAL ITAIPLFRVQ
 151 NYEVALLSVY VQAANLHLSI LRDVSVFGER WGYDTATINN RYSDLTSLIH
 201 VYTNHCVDTY NQGLRRLEGR FLSDWIVYNR FRRQLTISVL DIVAFFPNYD
 251 IRTYPIQTAT QLTREVYLDL PFINENLSPA ASYPTFSAAE SAIIRSPHLV
 301 DFLNSFTIYT DSLARYAYWG GHLVNSFRTG TTTNLIRSPL YGREGNTERP
 351 VTITASPSVP IFRTLSYITG LDNSNPVAGI EGVEFQNTIS RSIYRKSGPI
 401 DSFSELPPQD ASVSPAIGYS HRLCHATFLE RISGPRIAGT VFSWTHRSAS
 451 PTNEVSPSRI TQIPWVKAHT LASGASVIKG PGFTGGDILT RNSMGELGTL
 501 RVTFTGRLPQ SYYIRFRYAS VANRSGTFRY SQPPSYGISF PKTMDAGEPL
 551 TSRSFAHTTL FTPITFSRAQ EEFDLYIQSG VYIDRIEFIP VTATFEAEYD
 601 LERAQKVVNA LFTSTNQLGL KTDVTDYHID QVSNLVACLS DEFCLDEKRE
 651 LSEKVKHAKR LSDERNLLQD PNFRGINRQP DRGWRGSTDI TIQGGDDVFK
 701 ENYVTLPGTF DECYPTYLYQ KIDESKLKAY TRYQLRGYIE DSQDLEIYLI
 751 RYNAKHEIVN VPGTGSLWPL SVENQIGPCG EPNRCAPHLE WNPDLHCSCR
 801 DGEKCAHHSH HFSLDIDVGC TDLNEDLGVW VIFKIKTQDG HARLGNLEFL
 851 EEKPLLGEAL ARVKRAEKKW RDKRETLQLE TTIVYKEAKE SVDALFVNSQ
 901 YDRLQADTNI AMIHAADKRV HRIREAYLPE LSVIPGVNAA IFEELEERIF
 951 TAFSLYDARN IIKNGDFNNG LLCWNVKGHV EVEEQNNHRS VLVIPEWEAE
1001 VSQEVRVCPG RGYILRVTAY KEGYGEGCVT IHEIENNTDE LKFNNCVEEE
1051 VYPNNTVTCI NYTATQEEYE GTYTSRNRGY DEAYGNNPSV PADYASVYEE
1101 KSYTDRRREN PCESNRGYGD YTPLPAGYVT KELEYFPETD KVWIEIGETE
1151 GTFIVDSVEL LLMEE
```

C

Cry1Ab3

```
   1 MDNNPNINEC IPYNCLSNPE VEVLGGERIE TGYTPIDISL SLTQFLLSEF
  51 VPGAGFVLGL VDIIWGIFGP SQWDAFLVQI EQLINQRIEE FARNQAISRL
 101 EGLSNLYQIY AESFREWEAD PTNPALREEM RIQFNDMNSA LTTAIPLFAV
 151 QNYQVPLLSV YVQAANLHLS VLRDVSVFGQ RWGFDAATIN SRYNDLTRLI
 201 GNYTDHAVRW YNTGLERVWG PDSRDWIRYN QFRRELTLTV LDIVSLFPNY
 251 DSRTYPIRTV SQLTREIYTN PVLENFDGSF RGSAQGIEGS IRSPHLMDIL
 301 NSITIYTDAH RGEYYWSGHQ IMASPVGFSG PEFTFPLYGT MGNAAPQQRI
 351 VAQLGQGVYR TLSSTLYRRP FNIGINNQQL SVLDGTEFAY GTSSNLPSAV
 401 YRKSGTVDSL DEIPPQNNNV PPRQGFSHRL SHVSMFRSGF SNSSVSIIRA
 451 PMFSWIHRSA EFNNIIPSSQ ITQIPLTKST NLGSGTSVVK GPGFTGGDIL
 501 RRTSPGQIST LRVNITAPLS QRYRVRIRYA STTNLQFHTS IDGRPINQGN
 551 FSATMSSGSN LQSGSFRTVG FTTPFNFSNG SSVFTLSAHV FNSGNEVYID
 601 RIEFVPAEVT FEAEYDLERA QKAVNELFTS SNQIGLKTDV TDYHIDQVSN
 651 LVECLSDEFC LDEKKELSEK VKHAKRLSDE RNLLQDPNFR GINRQLDRGW
 701 RGSTDITIQG GDDVFKENYV TLLGTFDECY PTYLYQKIDE SKLKAYTRYQ
 751 LRGYIEDSQD LEIYLIRYNA KHETVNVPGT GSLWPLSAPS PIGKCAHHSH
 801 HFSLDIDVGC TDLNEDLGVW VIFKIKTQDG HARLGNLEFL EEKPLVGEAL
 851 ARVKRAEKKW RDKREKLEWE TNIVYKEAKE SVDALFVNSQ YDRLQADTNI
 901 AMIHAADKRV HSIREAYLPE LSVIPGVNAA IFEELEGRIF TAFSLYDARN
 951 VIKNGDFNNG LSCWNVKGHV DVEEQNNHRS VLVVPEWEAE VSQEVRVCPG
1001 RGYILRVTAY KEGYGEGCVT IHEIENNTDE LKFSNCVEEE VYPNNTVTCN
1051 DYTATQEEYE GTYTSRNRGY DGAYESNSSV PADYASAYEE KAYTDGRRDN
1101 PCESNRGYGD YTPLPAGYVT KELEYFPETD KVWIEIGETE GTFIVDSVEL
1151 LLMEE
```

D

Cry1Da_7

```
   1 MEINNQNQCV PYNCLSNPKE IILGEERLET GNTVADISLG LINFLYSNFV
  51 PGGGFIVGLL ELIWGFIGPS QWDIFLAQIE QLISQRIEEF ARNQAISRLE
 101 GLSNLYKVYV RAFSDWEKDP TNPALREEMR IQFNDMNSAL ITAIPLFRVQ
 151 NYEVALLSVY VQAANLHLSI LRDVSVFGER WGYDTATINN RYSDLTSLIH
 201 VYTNHCVDTY NQGLRRLEGR FLSDWIVYNR FRRQLTISVL DIVAFFPNYD
 251 IRTYPIQTAT QLTREVYLDL PFINENLSPA AVYPTFSAAE SAIIRSPHLV
 301 DFLNSFTIYT DSLARSAYWG GHLVNSFRTG TTTNLIRSPL YGREGNTERP
 351 VTITASPSVP IFRTLSYPTG LDNSNPVAGI EGVEFQNTIS RSIYRKSGPI
 401 DSFSELPPQD ASVSPAIGYS HRLCHATFLE RISGPRIAGT VFSWTHRSAS
 451 PTNEVSPSRI TQIPWVKAHT LASGASVIKG PGFTGGDILT RNSMGELGTL
 501 RVTFTGRLPQ SYYIRFRYAS VANRSGTFRY SQPPSYGISF PKTMDAGEPL
 551 TSRSFAHTTL FTPITFSRAQ EEFDLYIQSG VYIDRIEFIP VTATFEAEYD
 601 LERAQKVVNA LFTSTNQLGL KTDVTDYHID QVSNLVACLS DEFCLDEKRE
 651 LSEKVKHAKR LSDERNLLQD PNFRGINRQP DRGWRGSTDI TIQGGDDVFK
 701 ENYVTLPGTF DECYPTYLYQ KIDESKLKAY TRYQLRGYIE DSQDLEIYLI
 751 RYNAKHEIVN VPGTGSLWPL SVENQIGPCG EPNRCAPHLE WNPDLHCSCR
 801 DGEKCAHHSH HFSLDIDVGC TDLNEDLGVW VIFKIKTQDG HARLGNLEFL
 851 EEKPLLGEAL ARVKRAEKKW RDKRETLQLE TTIVYKEAKE SVDALFVNSQ
 901 YDRLQADTNI AMIHAADKRV HRIREAYLPE LSVIPGVNAA IFEELEERIF
 951 TAFSLYDARN IIKNGDFNNG LLCWNVKGHV EVEEQNNHRS VLVIPEWEAE
1001 VSQEVRVCPG RGYILRVTAY KEGYGEGCVT IHEIENNTDE LKFNNCVEEE
1051 VYPNNTVTCI NYTATQEEYE GTYTSRNRGY DEAYGNNPSV PADYASVYEE
1101 KSYTDRRREN PCESNRGYGD YTPLPAGYVT KELEYFPETD KVWIEIGETE
1151 GTFIVDSVEL LLMEE
```

Supplementary Figure 5.

A

Active core (D1-3)	Mutations in active core	PTX domain (D4-7)	Protein form tested	Insect	Slope	IC_{50} [µg/cm^2]	95% CI
Cry1Da_7	S282V,Y316S,I368P	Cry1Ab	full-length	CEW	4.03	0.063	0.051 – 0.078
Cry1Da	N/A	Cry1Ab	full-length	CEW	1.56	3.28	2.26 – 4.96

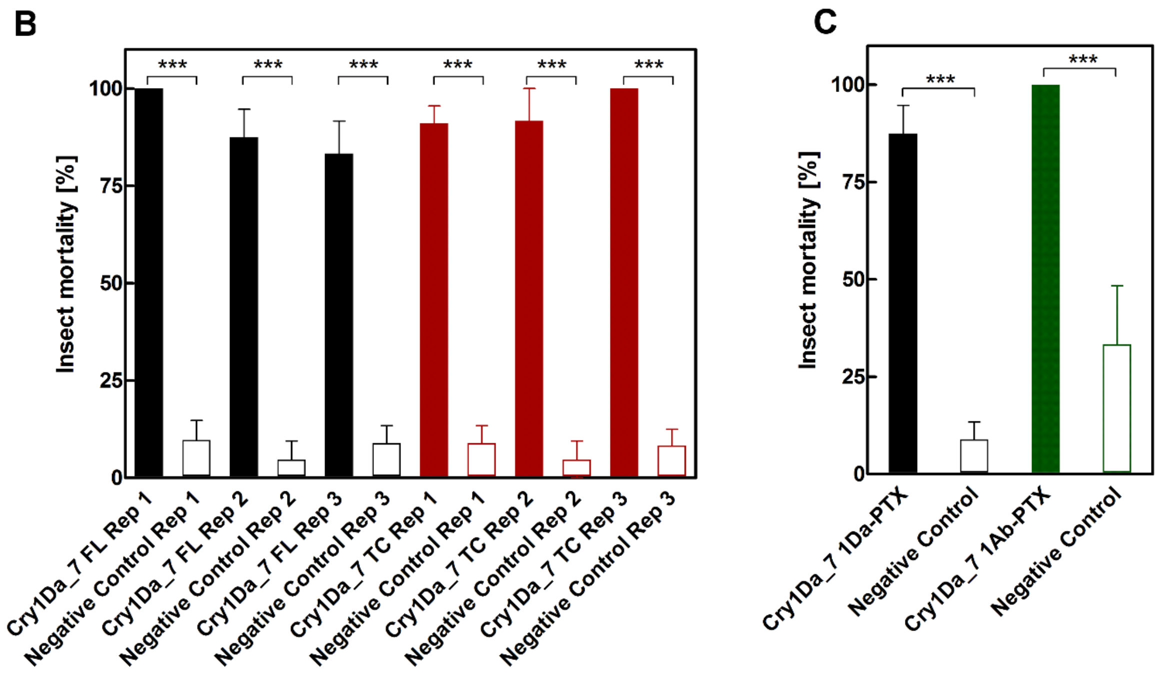

Supplementary Figure 6.

>Sf.APN9 c-His

MGLFRFVFLITIVSVTCKPVDLNIVQTEVDELVLEPESKNVINVTEGTSNEVTNNALVMR
NQVRPGMEVSSYAVEINANVEGGSFDGRAIVNLRIFDHDTREDPIVLNVAGLNINRVQY
AIMGGSNLFDADYSTDDDDQILEIEPGQLGSQYTLYIEYSGSLNGVGKGLYRGTYGDN
TYLAMNLHPTYARRVFPCMDEPTEASIISFSFEGLEYNHLTSNAMLEENSQTHFRPLVG
PPHLWGMIAHNFGTVNLPTSNVVLYARPGISNQESQASVAINFFFNNLNEWTQKPYFE
VIENQDGRMNIMVLPDVSTDWNSLSTVGIWEPYVLMEPVHAVKQRAIALTKIAEAMSR
QWFGYVIYPQNWRYEWVVAGFTTYSAYEMMRMFQADFATDINLLDVNTLFVTEVIQE
SLLRDAYINSNPLEPADDLFDEDAIRDHVNGLVKVKSAAIMRMIRLVLGDDDTDFIQIAA
RALLNMRSLETINTLKFIDGINSELVDSTVGDFDEYLDPWITSNGYPLLRVETTFSGVLL
HQTPFAFTNRESPSRLLPITYTTSIEKNFDNIHPQFMLDINHNLNVFLDEEDWILFNIQGQ
GYYRVTYDTVLWERIIEALEDPDRREEIHPLNRATLVDDALNLARAGQLGYDIAFSVVLS
MEHETEYAVWKAFVRNMNFLKKRLEAFVFDDDDLDPDIYLRMVRRTIPTFEREIGFYP
DTSINEPSMTSLTRGLVMDHACRAGYRPCIAAAVDWFYDPNNNEVVNPNIPHEIRPAV
YCTMVREGGEDAIEALLNRLEIEPTHYERVVILESLACSDDPDFINGYLMQTIVSNSPYS
VEERVKIFAAVAESSYTNADLARDFMRLRVNEIRNMYGVDKLEQLIYVLAENMADGDLT
REFEIWVQSSFNNLEDSQMTAERALAYVQENQNWGDKYTDDVYEWIDENHAHHHHH
HHHHH

>Sf.ABCb1

MKDSKGNAWQLKPGEKENENKLPDPLNEDVQKGQFSDTSSEPSEAKAEEVPSIPFITLFR

FASTRDKLFIICALICSAVAAVSTPLNTLLLAYLLEAMVNYSIFGDADAFMKSLLNFAIY
NAAVGAALVVLSYAATTLMNIAAYNQVYVIRQEYLKAALNQDFGYFDVHKTAEIANKMNS
DIMKLEEGIGEKLATFFFYQASFLSSVIMALVKGWKLALLCLISFPVTMTLVGVAGLIAA
SLSKKEAIATGKAGAIAEEVISAIRTVYAFSGQEKELDRYEGHLNDARKINVKKSLFNGL
AMGCLFFCIFCAYALSFWFGYRLMVTDGYDVSTMIAVFFGVMTGSANFGISSTLMEVFGS
ARGAGAHIFNMIDNVPTINPLQNRGTVPSDIEGNIELKNVEFHYPSRPDVPVLKGVSIKV
KRGQSVALVGHSGCGKSTIIQLISRFYDVVEGSVAIDGNDVRDLSVRWLREQIGLVGQEP
VLFNTTVRENIRYGRENATNEEIEACARQANAHQFIMKLPKGYDTLVGERGASLSGGQKQ
RIAIARALVRNPKILLLDEATSALDTSSEAKVQKALDKAQEGRTTIVVAHRLSTIRNVDV
IYVFKAGLVVECGNHTELMASKGHYYDMVMLQNLPGVDEQSPEKTKLSRETSIISEKDDE
DEFLEFRNDDKEEDAAEAPDISFMRVLKLNKPEWKSVTLASICSLMSGFCMPLFAVIFGD
FLALLDGDDPDEIQKGVSRLALIFVGIGVFSGITNFIVVFFYGIAGEALTKRLRLMMFRK
LLEMDIGFYDDKDNSTGALCARLSGEAAAVQGATGQRIGTVVQAVGTFGFALVLSLIFEW
RVGLVALTFVPIIIFVLYKEGRMTYAATSGTVKVMETSSKIAVEAVANVRTVASLGREET
FRREYSKQLRPALDTAVRSSHWRGLVFGMSRGVFNFVIASSLYYGGTIIVNEGVPFEEVF
KSAQALLMGATSAAQAFAFAPNFQKGIKAAGRVIVLLGRQSKITDPVEPAVKHFNGTGEA
SLQGIQFRYPTRPLVRVLKDLNLEIQRGKTVALVGASGCGKSTVIQLLERYYDPEDGIVA
QDGVPLPKLNLVDARRAIGFVQQEPILFDRTIGENIAYGNNEARVSSDEVIEAAKQANIH

NFITSLPLGYDTNIGSKGTQLSGGQKQRVAIARALIRRPKMLLLDEATSALDTESEKVVQ
EALDKAKAGRTCVMIAHRLSTVRDADVICVIHEGQVAEMGTHNELLELKGLYYNLNRRGY
A

>Sf.ABCc2 c-flag

MMDKSNKNTAANGNGGQRAGEPKERVRKKPNILSRIFVWWIFPVLITGNKRDVEEDDLIV
PSKKFNSERQGEYFERYWFEEVAIAEREDRDPSLWKAMRRAYWLQYMPGAIFVLLISGLR
TAQPLLFSQLLSYWSVDSEMSQQDAGLYALAMLGINFITMMCTHHNNLFVMRFSMKVKIA
ASSLLFRKLLRMSQVSVGDVAGGKLVNLLSNDVARFDYAFMFLHYLWVVPLQVGVVLYFV
YDAAGWAPYVGLFGVIILIMPLQAGLTKLTGVVRRMTAKRTDKRIKLMSEIINGIQVIKM
YAWEKPFQLVVKAARAYEMSALRKSIFIRSMFLGFMLFTERSVMFLTVLTLALTGNMISA
TLIYPIQQYFGIITMNVTLILPMAFASFSEMLISLERIQGFLLLDERSDIQITPKVVNGA
GSKLFNNSKKEGGLETGIVLPTKYSPTEANIARPMQDEPNMADYPVQLNKVNATWADLND
NKEMTLKNISLRVRKNKLCAVIGPVGSGKTSLLQLLLRELPVTSGNLSISGTVSYASQEP
WLFPATVRENILFGLEYNVAKYKEVCKVCSLLPDFKQFPYGDLSLVGERGVSLSGGQRAR
INLARAVYREADIYLLDDPLSAVDANVGRQLFDGCIKGYLSGKTCILVTHQIHYLKAADF
IVVLNEGSVENMGSYDELMKTGTEFSMLLSDQASEGSDTDKKERPAMMRGISKMSVKSDD
EEGEEKVQVLEAEERQSGSLKWDVLGRYMKSVNSWCMVVMAFLVLVITQGAATTTDYWLS
FWTNQVDGYIQTLPEGESPNPELNTQVGLLTTGQYLIVHGSVVLAIIILTQVRILSFVVM

TMRASENLHNTIYEKLIVAVMRFFDTNPSGRVLNRFSKDMGAMDELLPRSMLETVQMYLSLASVLVLNAIALPWTLIPTTVLMFIFVFLLKWYINAAQAVKRLEGTTKSPVFGMINSTISGLSTIRSSNSQDRLLNSFDDAQNLHTSAFYTFLGGSTAFGLYLDTLCLIYLGIIMSIFILGDFGELIPVGSVGLAVSQSMVLTMMLQMAAKFTADFLGQMTAVERVLEYTKLPTEENMETGPTTPPKGWPSAGEVTFSNVYLKYSPDDPPVLKDLNFAIKSGWKVGVVGRTGAGKSSLISALFRLSDITGSIKIDGLDTQGIAKKLLRSKISIIPQEPVLFSASLRYNLDPFDNYNDDDIWRALEQVELKESIPALDYKVSEGGTNFSMGQRQLVCLARAILRSNKILIMDEATANVDPQTDALIQKTIRKQFATCTVLTIAHRLNTIMDSDRVLVMDQGVAAEFDHPYILLSNPNSKFSSMVKETGDNMSRILFEVAKTKYESDSKTADYKDDDDK

>Sf.ABCc3

MDTKKGSKNDSKSKPPPPKPKSVPNVFKRTLFCWMLPIFYFGNRRDLEESDLPPPKNMYQSKMLGDKLERSWLKEEHEAKLAGRKPKFTKVLFKTFIWSYIPGGLMQATSIGLRTASPLLFSQLLRYWAADSPVDRETAMYYAISMILANWASSFMNHHGVLFCQQFGMKLRCAVGSLMFRKIMRMSNGSLGDTAAGKVVNILSNDLQRFDLSMVFLHYVWIIPLQIAAVIYLGYLQAGTAAFIGFAALIIIALPFQGGLGQYLGKIRLRTAEKTDNRIKIMSEVINGIQVIKMYAWEIPFQKVVGQKRAEELKEVKIATILRTVFLGFMMFTERAALFFTVLTYVLLGNVMSANVFYPLQQFMSAAQVNITLILPMVLSFTAELLVSLGRVEKFLLLEDRPDLKGHPEETSSNLFRNMSADGIPETNGSIRPLSYHRKSEASPIEPGSEIQLKVPKFTRSVSYQEDSALVLHDVSASWTGDPNMMALKNISMRLRKGKLCAIIGAVGSGKSSVLQLLLKELPSATGTISIYGKMSYACQEAWLFPSTVRENILFGLPFEPGKYKKVCRVCALEKDFKQFPYGDQTLVGERGVSLSGGQRARINLARAVYREADIYLLDDPLSAVDANVGRQLFEGCINGYLRGRT

RILVTHQIHFLKAADYIVVLNEGRIENMGTFDELVSSGKEFSMMLAQLQEGKEKDTESVGSRSIEEKEKPTLKTMISVNEGEEVQEFEAQKMKEEERQSGNLRWEVISAYFRSGGHVCFILFALLVVVLATTCAASVDYWVSYWTNQMAAYEESLGGESIEPGLDVQAGRFTVGQYLTIHGCLVAALVLMVNLRVFPFAYLCVSASAKLHNQMFSTMLRGVMRFFDTSSSGRILNRFTKDMGSLDEILPRTLLDVLQIYGTLTAILVLNAIALYWTLVPSAVLLVLFGFMVRIYLKAAQGIKRLEGTTKSPMFGTVTSSLSGISTIRASNAQERLIEQFDINQDLHTTSWNSYLNGGTTFGFYLDTMCLVYMTTIIFVFLFIDFGDAIPVGSVGLAVTQSNTLTLMLQHGARMLVEFLAQLTSVERILEYTRIDTEPDLFQGKVEMPPNWPYQGRIEFQNVSLRYAPNEQPVLKNLNIVIESGNKIGIVGRTGAGKSSLISALFRFAYLDGLISIDGLDTSLISRQGLRSKISIIPQEPILFSATIRYNLDPFDIYSDDDLWRALEQVDLKSAVPSLDFKVTEGGSNFSVGQRQLMCLARAVLRSNQILIMDEATANVDPQTDNFIQETIRRQFVSCTVLTIAHRLNTIMDSDKVLVMSSGQVAEYDHPYVLLSDPNSHFSAMVRETGEKNSANLFQVAKDAYFQSNLKENARDYKDDDDKX

>Sf.APN1 nested His

MASRWFNLLLGVILLQSVLAFGPIDVTDAHHHHHHEWIEYMGLINNPNYRLPTTTRPLHYKVRLQPNLDQDFELNGDVEINIKVESGNQPINEIKLHCQDMVINSLTVTSTTNTQVNLAQGTQFVCEETTSFLTIPTTTQLLNGNEYIIKISFVGKLQSGMRGFYRSWFFDENKQKRWMATTQFQPGHARQAFPCYDEPGFKATFDIILVRDDSLISLSNMPIRETITSTLYPQKKEDIYYTTPIMSTYLLAFIVADYKRIESGTNVNRPFHIYARGNVGDTGKYSLEVGEKLLTLMESYTQYNYYTMASHMEMKQAAIPDFSAGAMENWGLLTYREALILYDPENSNNFYKQRIANIISHEIAHMWFGNLVTCAWWDTLWLNEGFARYYQYYMTDKAEPHMGFKTRFIVEQLQMAMLSD

SFSNAHALTNPAVSDPDSVSNHFSTITYAKGASILRMTEHLLGGDTYEKGLREYLKKREFNTAEPKDLFESLDAAANADNSLADYDDMTIAKYFASWSEKAGHPLLTVHVDHASGRMTVVQTQFDVNSGVSSDNGLWHIPLTWTRAGNPEFENLKPSEFMSGPLKIINRGSTGREWVIFNKQQSGFYRVNYDATTWALLTQALRSNEREAIHEYNRAQIVDDVFVLARSNILSYTRALNILSFLEFEDKYAPWVAAITGFNFALRRLAHKTEEQQKLKDIIFKSSAAIIQRLGYTEASNADPLFMDNLLRMHLMTFLCNAGHAQCSQTGREYFKAWRESGTRIPPNMRPWVYCEGLRTGDLADFDYFWNRYVDEDLSNEKVVMIGAAGCTGNTEALHKFLSVIVDPKPTEQSIELIRPQDYSAAISSAVTSNEYNTMKVLEWLKDNPSHLQNGNGVSLLRSAASRLLNEADIFKVETWFATITSDEAIQAIKDGIATSNNNIKWYNSRVGEFSDYFETGYFDDLTGGSETPDPTTPEPTTAEPTTPEPTTPEPTTAEPTTAEPTTDESTPAEPTTAEPTTDEPEPGSANIASLSFFTLLVTLIINMV

>Sf.ABCa3 c-flag

MRLVPKQASPFAKFRLLMWKNFLQQWRHRTQTVLEILLPVLTMTLVLILRWQIEPAERETQTYPPFRANTLNFSTVVLFGLDCPNVSIAYSPTSPVLEDVVRNAITNLLIQNMEDLIARLPIEIELPPTIEINSTAILDWIKSRIRVQAYNNSHETRGIYIEEENTRRVIAVVEFDDKLYGAESLSNNLSYSLRFPERPRLNSLFQTGGRTWRTDAVFPVFETPGPRFSKSWEGGNDPGYVNEMFIALQQVISTELISRSTGVNMSEFTVFLQRYPHPPYIRDMALDLLQFMFPMFIMLSFSYTAINITRAVTVEKELQLKETMKIMGLPTWLHWTAWTCKQFVFLLVSASLTVILLKIN

WFTNEDGFSEYTVFTNTPWTVLMFFTMLYLTCVIFFSFMMSSFFSKASTAALCTVVVWFLTYIPAFLLAMDIEMSTTVQVFTCLSINSAMSYGFQLLLAKESTGGLQWGDFMSAPGTDSNRFVFGHVVIMLVVDCFVYMLVTLYFEQVMPGPFGTPKRWYFPFQLRFWFPHYKPGTVLVLENENSEFEDIIKEKEPNEHEVGVKMNNLTKIFGANTAVNNLSLNIYDDQITVLLGHNGAGKSTTISMLTGNLEVTRGTVTVAGYDMTHETYAARAHIGLCPQHNVLFNELTVREHLEFFARLKGFRGAELKSEIDTLIEKLELQDKRDYPSNGLSGGQKRRLCVGIALSGAARVVLLDEPTSGMDPSSRRALWELLQKEKKGRSMILTTHFMDEADILGDRVAIMAQGRLQCVGSPYFLKRHYGVGYTLVVVKDDDFDFEECTRLINKYIPDTVVKEDRGTEITYNLINDYSYAFEEMLNDLECNMEKIKYKNYGLTATTLEDVFMSVGSDLAPVNNSDNDDAVTTTTDSTIDDILKHELDSSLEELDRDESNVTGFRLLCQQVLAVWMKKGLTLIRSPWLMILQFFAPVILINATLGVMRYVMSLTPTIRSRFLSLTEGFTSTETLLSFNGTLGSSVGAIAAAAYEMMFTASDVENMGVTRIGNVPMDEYYLNRTMDPVVMGQLRHQILIGSTFDDNNATLWFSNFGYHDVAIALSTFHSAFLRAFNSTAQLNVYNHPLEATYRDQTDMQMMIAMLSMQLSSGIGSSVSIVSAVFIMFFIKERTSGAKLLQKAAGVQPAVLWGSAAVFNWAWFLITCVSIVITCAAFQVIGLSTAQELARMYLCVMLYGAAMLPLVYILSFAFNGPAVGFVGYYFMNVLFGMMGAQIVEALSSPQLNTAEAANILDYILQFFPLYSLITAVRFLNQVGLREYTCLQMCEYYQAVSPNLQCTMESLCSRNEECCVEPNVYFKWNQPGVSRYLTSMIISCIVFWTILMIIEYRVFQKLCTIKKTPPPLDESILDEDVQKEAQRARNVLPSQRYEHALIANDLSKYYGKHLAVNQISFGVNDGECFGLLGVN

GAGKTTTFKMLMGDESISSGEAFVSGHSVEKSLGKVHQNIGYCPQFDALFGELTGRETLH
MFAMMKGLRLRSAAPTAETLAHALGFLKHLDKRVNQYSGGTKRKLNTAIAFLGRTRLVFV
DEPTTGVDPAAKRHVWRATRGVQRAGRGVVLTSHSMEECEALCSRLTIMVNGRFQCFGTP
QHLKNKFSEGFHVNYGDYKDDDDK

>Sf.SR-C

MFIKCLLFLFVLSFIEADFYYPASTCPMRRIKNGRARPRQRGTFIKFSCSPRYTLVGNKY
ATCRFGQWDVPAPVCVKSGCSQLQEVKNSVNMTYHNNAWVVFFCLPGHQLIGSPVVYCDG
SNWNSTVPGCRDSSAKVSTECDFELPDLCGWKPDELHDFDWRRLNKKTPSSFLQTGPSYD
HTYGKNGSGYYMYIETTGRTENETARLLSPVYDAELAKNGCFIFYYHMYGRGMGGLRVYQ
KPDRVPMYQLLSSSKRNNYLLFEQWGDQGNEWYSSASMLTDVDDDFQIVIEGIRGNSFMS
DIAIDDVSIQRGENCTKAMLEATTPPAVLQESCVGRCDLYAEATGYRGCSCSIVCVVTQN
CCPDFLDVCDLDGISLDSDARGSSTAALPQTQKLIATTPDGNETELSDATTATTKVTVPT
TTSTKRPITTRSTTTSTTTKRPPIIIKRPTTTTKTTTVTKSTTTLSPRTTTAYPRTTTST
PKPTPKPTPKPTPKTTPKPTTTSSKPTTVTTSTVTVSSKPLTMPIEVSAVTEGTSMTTKQ
GTQEYLAKKSGGLSGATLAVTVIIAIAATFGLVWLAIVLTRSRGLAALARIRGRATSDPE
VHYLKSDVDDE

Supplementary Figure 7.

FAW proteins	Unique peptides identified [pcs]	Corresponding sequence coverage [%]
APN1	49	61
APN9	19	29
ABCb1	15	26
ABCa3	3	4
ABCc2	31	23
ABCc3	6	5

FIGURE LEGENDS

SUPPL. FIG 1. Amino acid sequence of the chimeric Cry1B.868 protein. Segments of the Cry1Be2, Cry1Ca1, and Cry1Ab3 sequences comprising Cry1B.868 are highlighted in red.

SUPPL. FIG 2. Concentration-response feeding assays demonstrating insecticidal activity of Cry1B.868 toward fall armyworm (FAW), *Spodoptera frugiperda*. Symbols are as follows, red circle, percent of dead insects; green square, percent of FAW larvae that have not molted to 2nd instar stadium; blue triangle, percent of FAW larvae that have not molted to 3rd instar stadium.

SUPPL. FIG 3. Specific insecticidal activity of the parent, Cry1Be2, and chimeric Cry1B.868 proteins on FAW.

SUPPL. FIG 4. Amino acid sequence of the chimeric Cry1Da.844 and Cry1Da_7 proteins. Segments of the Cry1Da1 and Cry1Ab3 sequences comprising Cry1Da.844 (A-C) as well as the three amino acid substitutions in Cry1Da_7 (D) are highlighted in red.

SUPPL. FIG 5. Specific insecticidal activity of the modified Cry1Da_7. (A) Assessment of the specific activity improvement against CEW via comparative analysis between Cry1Da and the modified Cry1Da_7, **(B)** FAW activity of 345 ng/cm^2 Cry1Da_7 before and after removing the protoxin domain via trypsin treatment, **(C)** FAW activity of the 345 ng/cm^2 Cry1Da_7 active core outfitted with Cry1Da vs. Cry1Ab protoxin domain (PTX).

SUPPL. FIG 6. FAW receptor sequences.

SUPPL. FIG 7. Proteomics data analysis indicating the presence of relevant *Bt* receptors in the FAW BBM sample. The columns indicate the protein ID, the number of unique peptides identified by proteomics and the corresponding percent of coverage in the full-length receptor sequence.
